# An Iterative, Pathologist-in-the-Loop Workflow for Generation of Clinical-Grade Synthetic Pathology Images in a Diverse Cohort of Pancreatic Tumors

**DOI:** 10.3390/cancers18183004

**Published:** 2026-09-16

**Authors:** Yixi Xu, Md Nasir, Valentina Matos-Romero, Tiane Chen, Ralph H. Hruban, William B. Weeks, Rahul Dodhia, Juan Lavista Ferres, Ashley L. Kiemen

**Affiliations:** 1 AI for Good Lab, Microsoft, Redmond, WA 98052, USAwbweeksmd@gmail.com (W.B.W.);; 2 Department of Chemical & Biomolecular Engineering, Johns Hopkins University, Baltimore, MD 21218, USA; 3 The Sol Goldman Pancreatic Cancer Research Center, Department of Pathology, Johns Hopkins University, Baltimore, MD 21218, USA; 4 Department of Oncology, The Johns Hopkins University School of Medicine, Baltimore, MD 21218, USA; 5 Department of Anatomy and Functional Evolution, The Johns Hopkins University School of Medicine, Baltimore, MD 21218, USA

**Keywords:** computational pathology, synthetic image generation, image tiling, pancreatic pathology, artificial intelligence

## Abstract

Background/Objectives: The training of diagnostic pancreatic pathologists is largely limited by the diversity of available pathology images, especially those of rare diseases or conditions. Methods: Using a cohort of seven pancreatic neoplasms, we developed an iterative, pathologist-in-the-loop workflow integrating scalable tile pruning, generative-model optimization, and postprocessing truncation. Two pathologists evaluated synthetic-image quality and subtype representation on a 0–3 scale; the independent pathologist was blinded to image source and truncation condition and also rated curated real training tiles. Results: Untruncated images had lower class-balanced FID than per-class-truncated images (6.64 versus 29.08) and higher recall and coverage, whereas truncation increased precision. The independent pathologist rated per-class-truncated images higher than matched non-truncated images (mean difference 0.80, bootstrap 95% CI 0.57–1.03). Quadratic-weighted Cohen’s κ was 0.599 (95% CI 0.489–0.691); after grouping ratings as 0–1 versus 2–3, raw agreement was 80.7% (95% CI 75.0–86.4%). Conclusions: Per-class truncation improved independently rated image quality and subtype representation. Successful synthetic histology generation requires careful data curation, domain-specific oversight, and independent validation; clinical utility requires separate task-based evaluation.

## Simple Summary

The training of diagnostic pancreatic pathologists is largely limited by the diversity of available pathology images. This study developed a workflow combining automated image processing with iterative feedback from expert pathologists to generate realistic, clinical-grade synthetic images of pancreatic tumors, with a focus on rare subtypes, to enrich educational cohorts. Quantitative evaluation showed that truncation increased precision while reducing recall and coverage, consistent with a quality-diversity trade-off. In an independent review blinded to image source and truncation condition, per-class truncation improved ratings of image quality and subtype representation relative to no truncation. After grouping ratings as 0–1 versus 2–3, the pathologists agreed on 80.7% of classifications, although agreement on the exact four-level score was lower. These results emphasize the importance of careful data selection, domain expertise, and independent multi-reader validation.

## 1. Introduction

The field of diagnostic anatomic pathology is an inherently visual field. Microscope slides are prepared from biopsies and resections, stained using hematoxylin and eosin (H&E), and examined by trained pathologists who establish diagnoses that determine patient treatment and prognosis. Training a qualified diagnostic pathologist takes years of exposure to a wide variety of organs and diseases. This educational process requires large numbers of images that represent the full spectrum of a disease’s presentation. However, many training programs do not have access to the necessary images, especially for rare diseases of the pancreas that are infrequently seen. Generation of synthetic images using artificial intelligence (AI) has the potential to overcome this challenge by increasing the number of images available for pathology education, as well as for other image-intensive fields of medicine such as radiology.

Further, it has long been understood that AI models learn biases when trained on unbalanced datasets [1,2]. AI practitioners use a variety of techniques, including undersampling of majority classes, oversampling of minority classes, and data augmentation to account for unbalanced cohorts. However, this problem is often overlooked in training of large language and foundation models, where massive datasets are required for state-of-the-art performance. Generation of a perfectly balanced cohort is often sacrificed in favor of maximization of training dataset size [3,4].

In pathology, this translates to models trained with different numbers of images per organ, disease, or cell type, which can lead to underperformance on rare diseases or populations that are less likely to exist in digitized archives, such as those related to pancreas pathology [5,6,7]. We hypothesize that this imbalance leads to uneven performance based on an entity’s representation in the training dataset and propose generation of clinical-grade synthetic pathology images as one potential approach to this problem. One approach used by researchers to avoid discarding images from oversampled classes is to generate synthetic images of undersampled classes [8,9]. Here, we present a workflow for generating synthetic pancreatic pathology images designed to depict subtype-associated morphology in isolated image tiles.

The size of pathology images and the sparsity of diagnostic morphological features constrain our ability to design precise AI models for generating clinical-grade synthetic images. Pathology slides are scanned at high magnification to enable visual differentiation of cellular morphology for clinical evaluation, resulting in the generation of whole slide images (WSI) whose dimensions can exceed 100,000 × 100,000 pixels. As such, AI models trained on WSIs must incorporate tiling, where images are cropped to dimensions that are reasonable for GPU memory handling. This technical constraint introduces errors in AI models, as, in practice, very few tiles in a WSI contain the morphological features relevant for diagnosis. In classification problems, this has been mitigated using multiple instance learning, where an aggregate decision is made from cumulative inference on many tiles [10,11]. However, for synthetic image generation, the problem persists.

**Figure 1:**
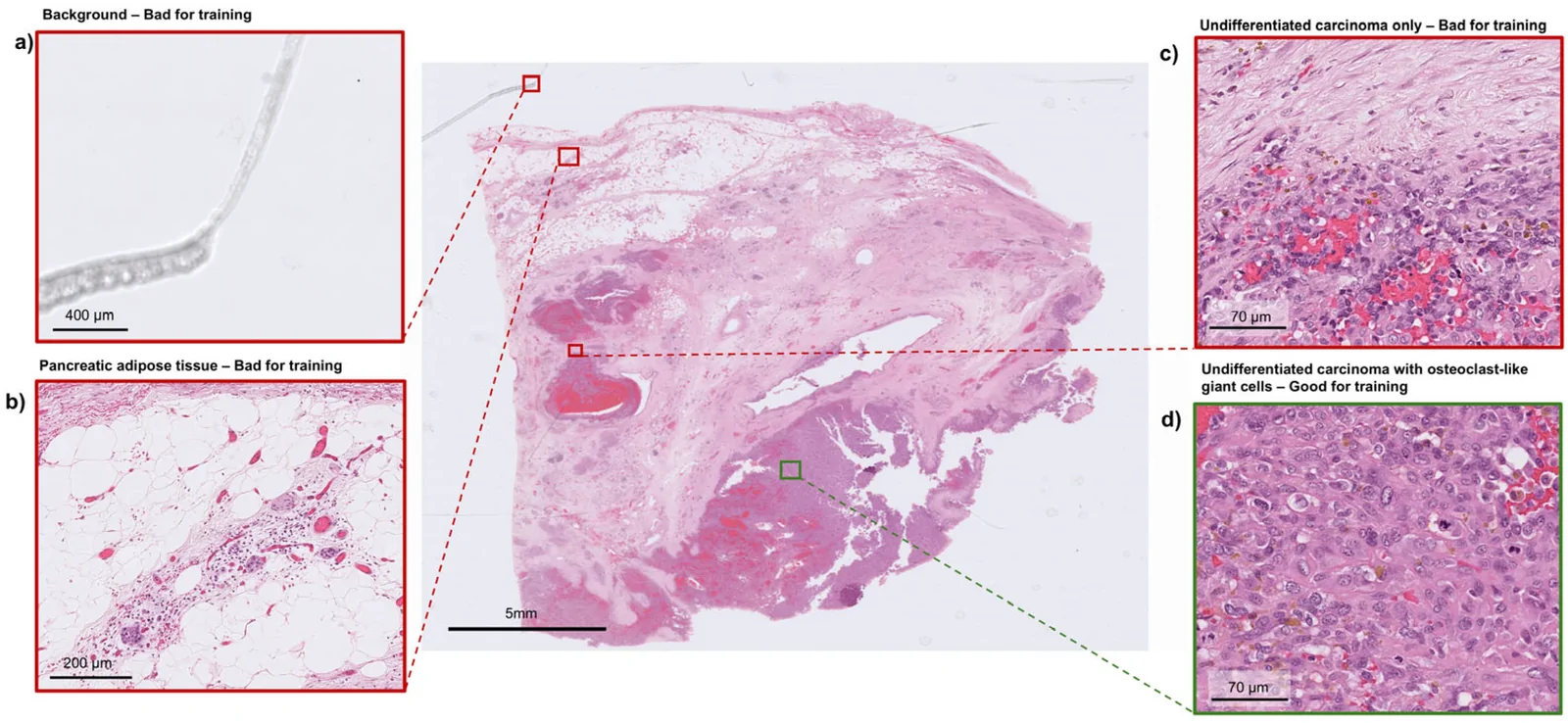
Tile pruning is necessary in digital pathology applications. A sample whole slide image containing hematoxylin and eosin-stained pancreatic tissue from a patient diagnosed with undifferentiated carcinoma with osteoclast-like giant cells of the pancreas. The image, when cropped into tiles, contains many regions that are not beneficial for model training, including tiles comprised solely of (**a**) background, (**b**) non-neoplastic parenchyma, (**c**) tumor regions that do not contain all necessary diagnostic criteria (here, undifferentiated carcinoma is present but osteoclast-like giant cells are absent). Tiles beneficial for model training contain in-focus areas possessing all morphological criteria necessary for clinical diagnosis, as depicted in (**d**).

For example, undifferentiated carcinoma with osteoclast-like giant cells is a rare form of pancreatic cancer that requires two morphologies for definitive diagnosis (Figure 1). Many regions of the tumor contain neither or only one of these morphologies, leading to poor model performance if these regions are not pruned from the training dataset. While numerous methods exist for detection of background and artifacts [12], removing technically high-quality tiles that do not contain the target combination of subtype-associated morphology is more challenging and requires a pathologist-in-the-loop approach.

### 1.1. Related Work: Tile Selection for Deep Learning Model Training

The processing of gigapixel WSIs for deep learning requires robust tile selection and pruning strategies to manage extreme computational overhead and the high prevalence of non-representative regions. A critical initial step in this process is background pruning, which separates tissue from the surrounding glass slide. Traditional approaches frequently utilize thresholding techniques within grayscale or hue-saturation-value color spaces. These methods employ either predefined heuristics or automated algorithms, such as Otsu’s method, to determine the threshold to separate foreground tissue from the background based on pixel intensity distributions [13,14]. Alternatively, more recent workflows formulate tissue segmentation as a supervised learning task, leveraging deep learning architectures to generate precise binary masks that exclude non-tissue areas [12].

Beyond background removal, the exclusion of low-quality tiles is essential to ensure that the downstream models do not learn or reproduce slide preparation and scanning artifacts. These include out-of-focus regions, pen markings, tissue folds, air bubbles under a coverslip, and debris. Existing literature includes two types of tools: those utilizing traditional hand-crafted features to quantify sharpness and color consistency [15], and those employing deep learning to automate the detection of specific artifacts [12,16].

While pruning removes noise, selecting tiles that contain the morphological features relevant for diagnosis remains a challenge. Current generative frameworks [17,18,19] for digital pathology rely heavily on high-fidelity human annotations to exclude areas that do not contain the target subtype morphology. However, high-quality manual data annotation is labor-intensive and lacks scalability, particularly in subtypes characterized by low cellularity.

To the best of our knowledge, this work is the first to propose a framework to address the selection of representative tiles under low-resource annotation constraints. Though not studied in the field of generative models, this objective intersects with “informative” patch selection strategies studied in weakly supervised learning. For example, attention-based multiple instance learning frameworks use attention mechanisms to automatically rank tiles based on their contribution to the slide-level diagnosis [14,20]. Similarly, diversity-driven methods utilize feature-space clustering (e.g., K-means) to sample patches from different regions of the latent space [21,22]. While these methods effectively capture the morphological heterogeneity of a slide, they are fundamentally optimized for discriminative multiple instance learning architectures. Such models exhibit a significantly higher tolerance for noise and non-representative instances, as the aggregation functions can effectively suppress outliers. In contrast, generative modeling is highly sensitive to input quality; training on non-representative or noisy tiles often leads to the synthesis of artifacts or non-tumor areas.

### 1.2. Related Work: Synthetic Image Generation

Generative Adversarial Networks (GANs) have long served as the cornerstone for medical image synthesis due to their ability to learn complex high-dimensional distributions through an adversarial min-max game [23,24,25]. However, GANs are often plagued by mode collapse, where the generator produces a limited variety of samples, especially for minority classes, and unstable training dynamics that can lead to artifacts [26]. Recent advancements [25,27,28,29] have attempted to mitigate these issues through architectural changes and training refinements for high-resolution synthesis.

Recently, diffusion models have surpassed GANs in various image generation tasks [30]. However, the iterative nature of the reverse diffusion process remains computationally intensive [30,31]. These models operate through a stochastic process of gradually adding Gaussian noise to an image and subsequently learning a reverse denoising transition to recover the original data distribution [32]. Furthermore, they introduce their own unique challenges for clinical deployment. Recent studies have highlighted a higher risk of training data memorization in diffusion architectures compared to GANs, posing significant privacy concerns when models are trained on sensitive patient data [33].

In computational pathology, generative models have been utilized to address data scarcity, particularly through the augmentation of minority classes and the synthesis of rare pathological variants [34,35,36,37,38]. These frameworks have also found utility in educational applications, enhancing the training of pathologists through the generation of diverse educational cohorts [17]. As deep learning architectures move toward more complex diagnostic and prognostic tasks, synthetic datasets serve as a critical resource for improving model generalizability and robustness [39,40].

Beyond simple augmentation, generative techniques have the potential to facilitate virtual staining [41] and super-resolution tasks [42] aimed at enhancing the spatial fidelity of high-resolution datasets. This is particularly relevant for 3D tissue mapping, where generative models can perform volumetric interpolation to reconstruct continuity in skipped or corrupted sections. Despite these advancements, the application of generative synthesis remains largely focused on common coarse-grained malignancies. To the best of our knowledge, this work represents the first effort to use generative models for the synthesis of fine-grained pancreatic tumor subtypes.

Here, our goal was to generate high-quality synthetic image tiles of pancreatic tumors that represented subtype-associated morphology as assessed by pathologists. We utilized a cohort containing seven types of pancreatic tumors with diverse morphological presentations and developed an iterative workflow for clinical-grade synthetic image generation via tile pruning and pathologist review. Our results show that nuanced pruning of biologically ambiguous tiles from the training dataset supports the generation of images with stronger subtype representation. This approach provides a preprocessing foundation for future studies of pathology.

## 2. Materials and Methods

In this section, we introduce methods for tile generation and pruning from large gigapixel images. Following that, we describe StyleGAN2, a previously published method for synthetic image generation. Finally, we describe our approach for iterative model finetuning using postprocessing truncation and a workflow for pathologist-in-the-loop feedback.

### 2.1. Data Acquisition

**Figure 2:**
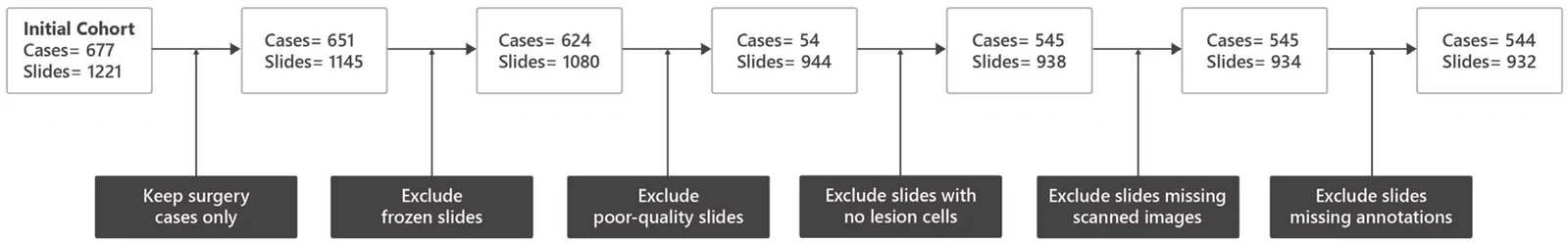
Flowchart of data inclusion and exclusion.

We retrospectively collected 1221 digitized H&E-stained tissue sections taken from 677 pancreatic resections performed at the Johns Hopkins Hospital for one of seven types of pancreatic tumors. All whole slide images were reviewed by a pathologist who rendered both primary and, where applicable, secondary pathological diagnoses. Regions of interest corresponding to the primary diagnosis, such as neoplastic cells, were annotated by trained personnel at low magnification. For the remainder of this study, we refer to these regions as low-resolution annotations. Following quality assessment to exclude images of overall poor diagnostic quality due to severe artifacts or that did not contain neoplastic cells, the final cohort consisted of 932 images from 544 resections (Figure 2). Table 1 summarizes the number of resections and images for each tumor type, with a representative tile for each.

### 2.2. Pathology Image Tile Pruning

**Figure 3:**
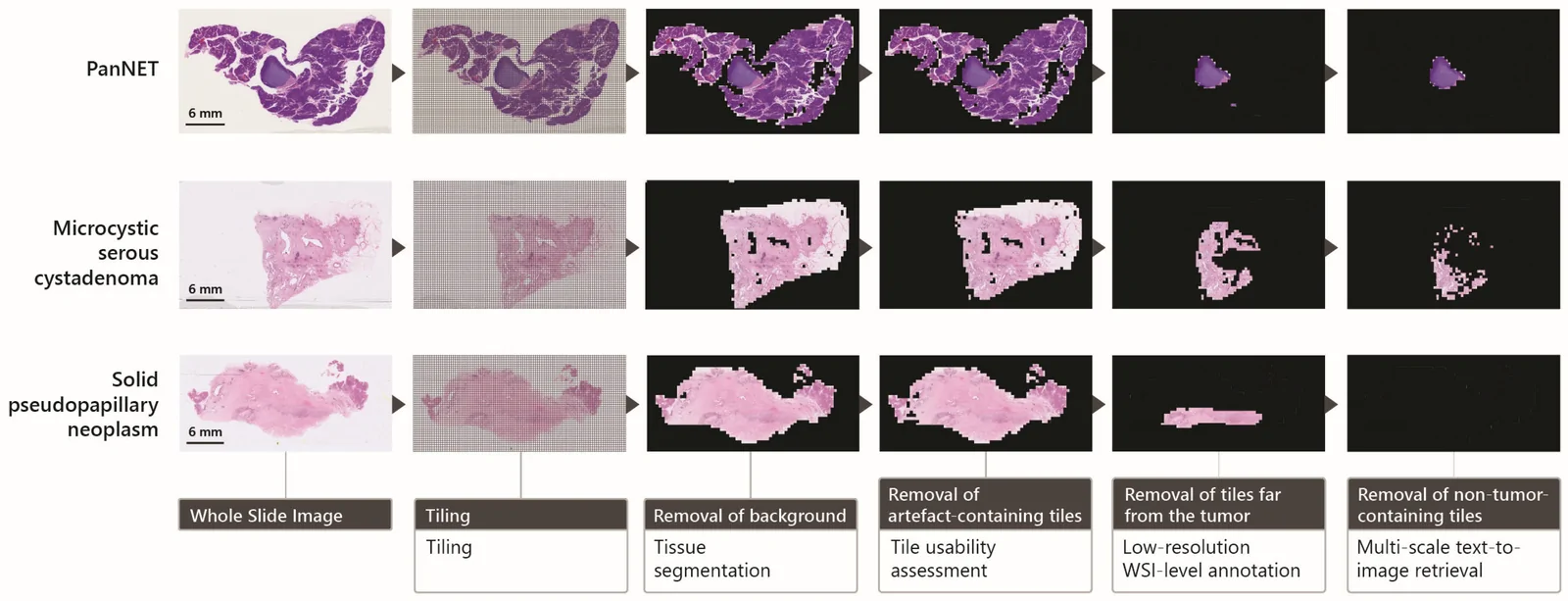
Data preprocessing pipeline for three whole slide images. Whole slide images were cropped into tiles. Tiles identified as primarily background were excluded. Next, tiles containing low-quality tissue were excluded. Next, tiles outside of the expert-drawn manual annotation were excluded. Finally, tiles predicted not to contain the primary diagnosis of the patient were excluded. This left tiles of high-quality tissue regions containing the diagnostic tumor cells.

WSIs with a resolution of 0.5 mpp (corresponding to 20× magnification) were cropped into tiles of dimensions 1024 × 1024 with a stride of 512 and saved as uncompressed .PNG files. Pathology WSIs contain regions of high-quality tissue, low-quality tissue (containing artifacts such as tissue damage or out-of-focus), and empty background with no tissue present. Within high-quality tissue are undesired regions of normal pancreatic parenchyma, stroma, and fat, as well as desired regions containing one or more diagnostic features. We designed a multi-step workflow to isolate high-quality tiles containing the diagnostic features of each pancreatic tumor type present in our cohort (Figure 3).

#### 2.2.1. Removal of Background and Artifact-Containing Tiles

To remove tiles containing majority background, out-of-focus, or damaged tissue, we employed a two-step approach. First, we applied an open-source tissue segmentation model [12] to determine the percentage tissue composition of each tile, removing tiles with <50% tissue composition.

**Figure 4:**
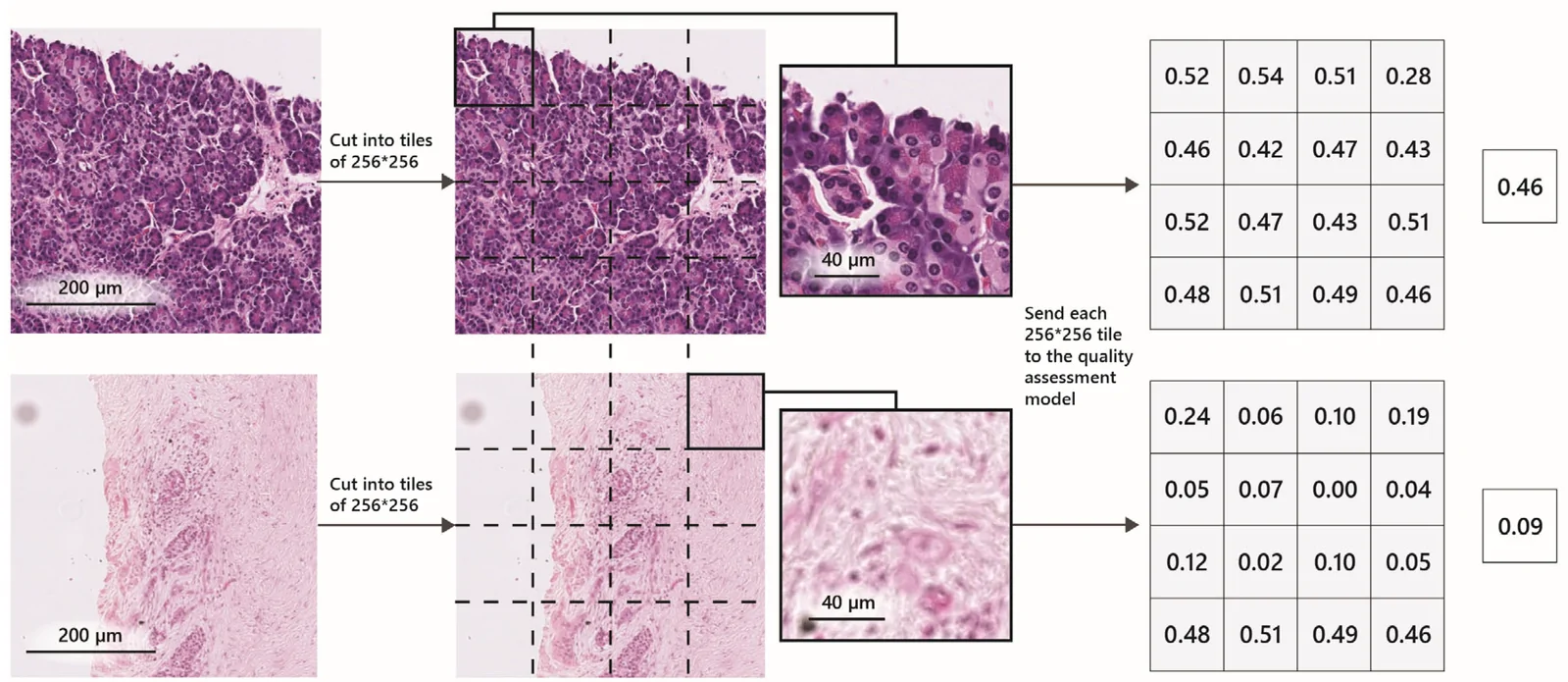
Sample utility score estimation for two 1024 × 1024 tiles, one found to be of adequate quality, and one found to be of poor quality.

Next, we cropped each remaining 1024 × 1024 tile into 16 non-overlapping sub-tiles of 256 × 256 and utilized an open-source pathology image quality assessment model to estimate its utility score [12]. The utility score ranged from 0 to 1, where higher scores indicate better tile quality. We defined an aggregate utility score for each tile as the average score of its constituent sub-tiles (Figure 4). We then filtered out low-quality tiles as those with a mean utility score less than or equal to 0.22 (See Supplementary Information for threshold optimization details).

#### 2.2.2. Removal of Tiles Far from the Tumor via Low-Resolution WSI-Level Annotations

We incorporated low-resolution annotation of the tumor region on each WSI to remove non-target tiles that were missed by the previous approaches. These low-resolution annotations were performed by a trained, non-pathologist professional. Given the impracticality of high-resolution manual annotation of tumor cells in thousands of tiles making up gigapixel whole slide images, we selected a slide-level, low-resolution annotation strategy instead of a tile-level annotation. As our estimates in Table 2 show, a full tile-level annotation would require over 7100 h, whereas we completed the slide-level annotation in approximately 20 h.

We also considered an alternative segmentation strategy. This would involve annotating tumor/non-tumor areas (or cyst/non-cyst areas for cystic subtypes) to train a separate segmentation model for each subtype [43,44]. However, scaling this supervised approach beyond our seven selected subtypes to the full spectrum of structures histologically visible in this unique cohort was resource prohibitive. Therefore, we chose the slide-level approach as the most practical method to label the entire cohort.

#### 2.2.3. Removal of Non-Tumor-Containing Tiles Using Multi-Scale Text-to-Image Retrieval

After background, artifact, and annotation-based filtering, we used the pretrained CONtrastive learning from Captions for Histopathology (CONCH) model [45] to remove tiles that lacked morphology associated with the primary diagnosis. We did not update the model weights. Candidate text classes included the primary diagnosis, normal tissue components, secondary diagnoses, and alternative class names or synonyms. Starting from this baseline lexicon, we reviewed false-positive retrievals and retained terms that improved F1. Because low-resolution annotations could incompletely represent sparse or heterogeneous tumor morphology, pathologist review was also used to identify clinically relevant descriptors not captured by the quantitative comparison. Retrievals from synonymous prompts were ensembled.

For multiscale retrieval, each 1024 × 1024 tile was evaluated at low magnification after downsampling from 0.5 to 1.0 mpp and at the native 0.5 mpp by dividing it into four non-overlapping 512 × 512 subtiles. The low-magnification view provided a prediction for the full tile. At high magnification, the parent tile was considered positive when at least one subtile was classified as the primary diagnosis. A tile was retained only when the low- and high-magnification predictions concordantly identified the primary diagnostic category.

#### 2.2.4. Removal of Slides Using Recall-Validated Annotation Pruning

**Figure 5:**
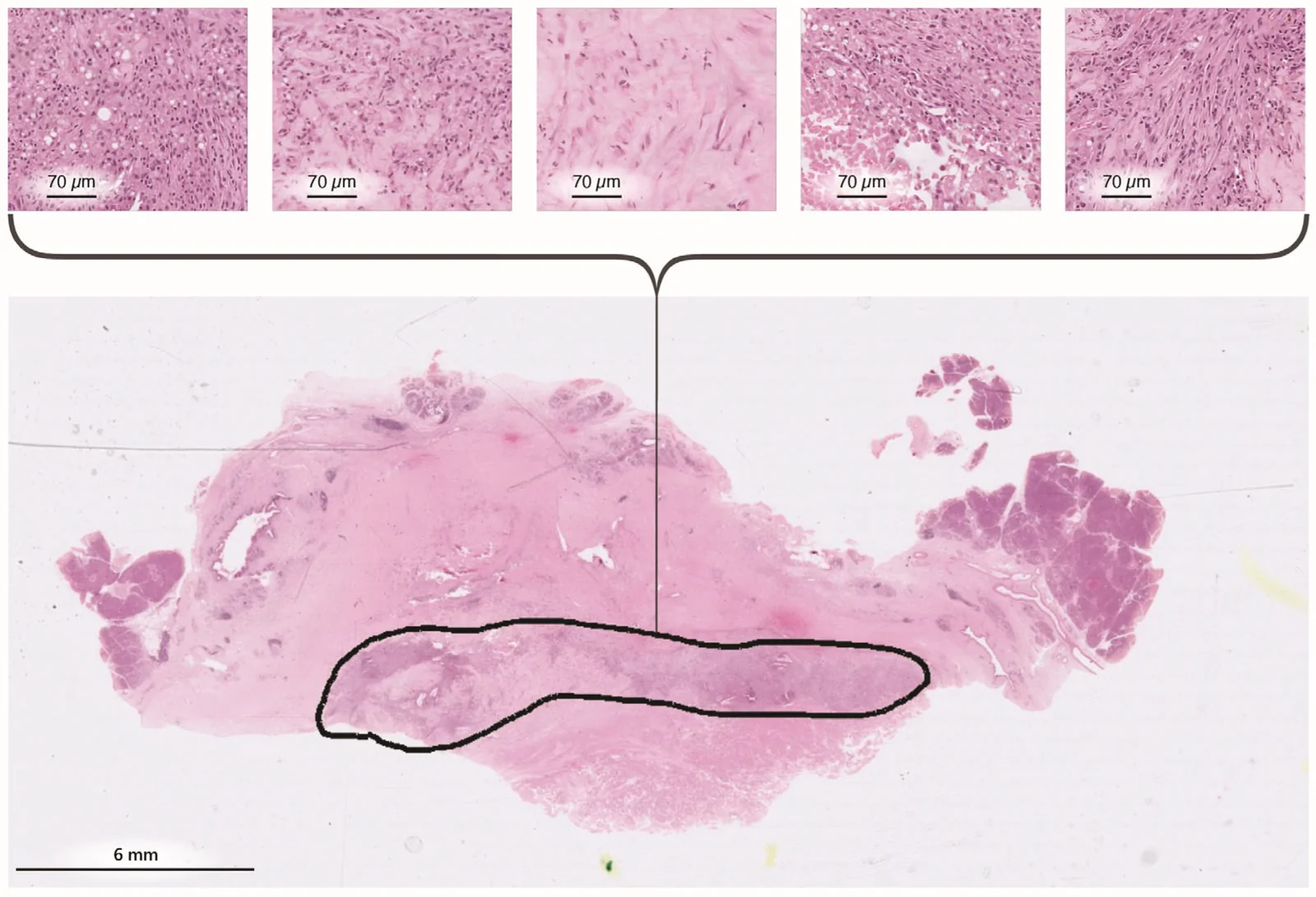
Examples of misrepresentative tiles sampled from low-resolution solid pseudopapillary neoplasm (SPN) annotations. These tiles were extracted from regions manually annotated as SPN. However, this tumor was infarcted (necrotic) and lacks the diagnostic histological features of the neoplasm, illustrating the inherent label noise in tile-level datasets derived from low-resolution annotation.

Because diagnoses were assigned at the case level, a multi-slide case could include individual slides in which the primary tumor was absent or no longer recognizable, including sections dominated by infarcted tissue (Figure 5). Applying the case-level diagnosis directly to every slide could therefore introduce slide-level label discordance. We used annotation recall as a slide-level label-fidelity quality-control heuristic. For each slide, annotation recall was defined as the proportion of tiles within the manual annotation that the multiscale retrieval model classified as the case’s primary diagnosis. Slides with annotation recall <0.50 were excluded from generative-model training. The 0.50 cutoff was chosen empirically and was not tuned or optimized against a quantitative outcome. Its purpose was to identify slides whose annotated tissue was poorly concordant with the case-level diagnosis, such as slides without the primary tumor or with predominantly infarcted, non-diagnostic tissue.

### 2.3. Model Training and Optimization

#### 2.3.1. Discriminative Preprocessing Configuration and Robustness Evaluation

Trained discriminative models for pathology image quality assessment and multiscale text-to-image retrieval were used to configure which tiles entered generative-model training. Neither model’s weights were updated on this cohort. The final configuration was selected using all annotated data to curate the generative model training set. We separately evaluated utility-threshold and prompt-template selection using case-disjoint five-fold cross-validation, with all observations from a case assigned to one fold and uncertainty estimated using bootstrapping. Detailed protocols and results are provided in Section 1 (Table S1, and Figure S1) and Section 2 (Table S4) of the Supplementary Materials, respectively.

#### 2.3.2. Generative Model Training

We selected conditional StyleGAN2 because prior studies demonstrated high-resolution pathology image synthesis [17,18] and because its single-pass generation is computationally efficient relative to iterative diffusion sampling [30]. This efficiency enabled repeated experiments under limited compute. We fine-tuned StyleGAN2 [25] on the curated training tile dataset using a batch size of 16 and input and intermediate latent dimensions of 1024. Adaptive discriminator augmentation was used to improve training stability. To reduce overrepresentation of cases contributing many tiles, we used case-ID oversampling so that cases contributed more equally. Model training used four NVIDIA V100 GPUs. This configuration was designed for efficient 1024 × 1024 pathology-tile synthesis.

#### 2.3.3. Post-Processing Truncation

To mitigate the generation of tiles without the diagnostic features of each tumor present, we applied a truncation strategy in the intermediate latent space of StyleGAN2 [25]. We first compute $\bar{w}$, the center of mass of the intermediate space, which represents an average latent variable (a sort of an average pathology image). We then scale the deviation of a given latent intermediate variable w from the center as $w=\bar{w}+\psi (w\;-\;\bar{w})$, where the truncation factor ψ ≤ 1. A ψ value of 1.0 returns the original intermediate variable, thus applying no truncation. We used a ψ=0.5 for the truncation. In addition, we computed the center of mass $\bar{w}$ using two different methods. The first is a per-class approach, where a unique $\bar{w_{c}}$ was calculated for each subtype c, representing the average latent profile for that specific class. The second is a class-agnostic approach, where a single, universal $\bar{w}$ was calculated from all subtypes combined, representing a global average latent profile.

#### 2.3.4. Quantitative Evaluation of Synthetic-Image Fidelity and Diversity

We evaluated synthetic-image quality and diversity using FID and KID [46,47], precision and recall [48], density and coverage [49], and Inception Score (IS) [26]. FID and KID measure distributional similarity to real images, with lower values indicating better agreement. Precision and density measure fidelity and concentration in realistic feature-space regions, with higher values indicating better fidelity. Recall and coverage measure diversity and representation of the real-image distribution, with higher values indicating better coverage. We treated IS as exploratory because it relies on an ImageNet classifier that is not pathology-specific, although higher values conventionally indicate better quality and diversity. We compared matched-seed per-class-truncated (ψ = 0.5) and untruncated synthetic image sets, each containing 35,000 images (5000 per subtype).

### 2.4. Pathologist-in-the-Loop Iteration and Image-Quality Assessment

**Figure 6:**
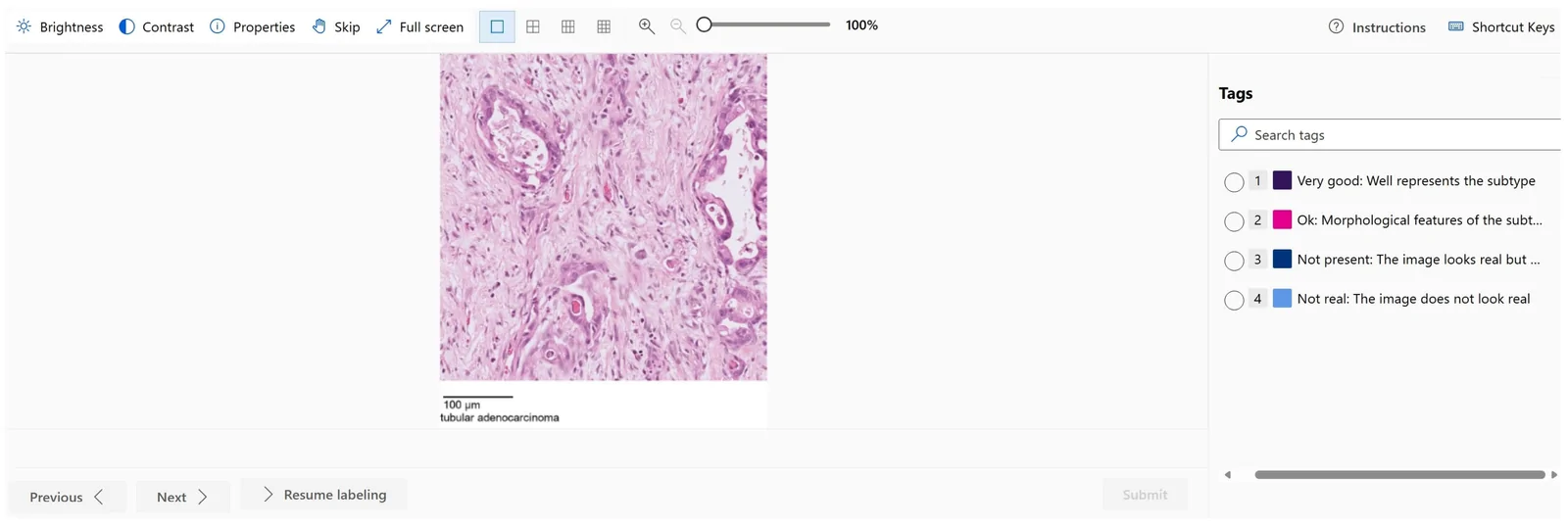
Image labeling interface. A screenshot of the Azure Machine Learning labeling tool used by the pathologist to grade synthetic images.

To evaluate synthetic image quality, two pathologists conducted blinded reviews using a 4-point scale (Figure 6). The ratings were 3 (very good: well represents the subtype), 2 (ok: morphological features of the subtype are present, but there are issues such as cut-offs or regions of low quality), 1 (not present: the image looks real but does not represent the specified subtype), or 0 (not real: the image does not look real). This tile-level scale assessed image quality and representation of the specified subtype. The original pathologist, who participated in iterative model development, rated 10 images per subtype under each generation setting while blinded to the specific generation parameters. An independent pathologist, who had no role in model development, subsequently evaluated the same 140 synthetic images (70 per-class-truncated and 70 non-truncated) alongside 70 real tiles. The real tiles were drawn from the final training tile set by randomly sampling 10 cases per subtype without replacement and selecting exactly one tile per sampled case. For the independent review, all 210 images were randomly interleaved, concealing both source (real or synthetic) and truncation condition. We report rater-specific mean scores with 95% bootstrap confidence intervals for each subtype and condition. The independent-rater condition effect was tested using a two-sided paired permutation test with 200,000 within-pair label swaps. Inter-rater agreement on the 140 shared synthetic images was quantified using quadratic-weighted Cohen’s κ and ICC (2,1), with bootstrap confidence intervals.

## 3. Results

We evaluated the impact of retrieval and postprocessing design choices on synthetic image generation from a dataset of pancreatic histology images covering seven histologically distinct pancreatic tumor types. Here, we present the results of this workflow and several technical optimizations we performed to improve the performance of our model.

### 3.1. Text-to-Image Retrieval Can Be Improved Using Expanded Lexicon

Our text-to-image retrieval process began by establishing a baseline list including normal, primary diagnosis, and any secondary diagnosis if available. This lexicon was then iteratively refined to the expanded lexicon (see the full lexicon in Tables S3, S5 and S6).

**Figure 7:**
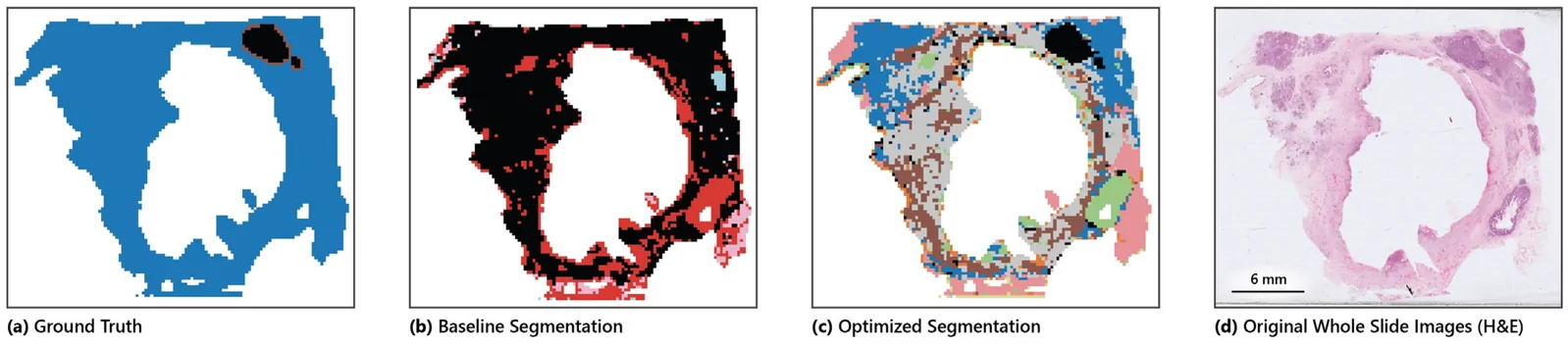
Lexicon optimization dramatically improves our ability to segment the tumor region in whole slide images with extensive over-calling of well-differentiated pancreatic neuroendocrine tumor and intraductal papillary mucinous neoplasm using the baseline lexicon. The well-differentiated pancreatic neuroendocrine tumor is consistently represented by the black segmented region across the ground truth segmentation, the baseline segmentation, and the optimized segmentation.

We compared the retrieval performance of our lexicon versus a baseline of only including primary diagnosis class names with no primary diagnosis synonyms, secondary diagnosis class names or ‘normal’ cell types. Our method improved the overall F1 score from 0.55 (baseline) to 0.64. As shown in Figure 7 and Table 3, this performance gain, primarily driven by increased precision, was observed across all subtypes except for solid pseudopapillary neoplasm (Table 4). Although a concurrent decrease in recall was noted, we attribute this in part to the limited precision of the ground-truth labels (Figure 5). These labels were derived from low-resolution, whole-slide annotations, which may inaccurately represent non-focal pathologies, such as diffuse tumors or cysts, that lack dense morphological features at the tile level.

Prompt-template variability and case-disjoint prompt-selection robustness are reported in Section 2 of the Supplementary Material, with fold-specific results in Table S4 and all 49 candidate templates in Table S3.

### 3.2. Quantitative and Pathologist Evaluation of Synthetic Images

#### 3.2.1. Quantitative Fidelity and Diversity Metrics

The untruncated condition showed better class-balanced distributional agreement and diversity than per-class truncation, with lower FID (6.637 versus 29.084) and KID (0.00328 versus 0.01678), higher recall (0.460 versus 0.104), and higher coverage (0.781 versus 0.606; Table 5). Conversely, per-class truncation increased precision (0.743 versus 0.631) and density (1.348 versus 0.775), indicating that generated images were more concentrated within narrower high-density regions of the real-image feature space. The same direction was observed for every subtype: ψ = 1.0 had lower FID and KID and higher recall and coverage, whereas ψ = 0.5 had higher precision and density (Table S9). Thus, truncation concentrated the generated distribution at the cost of measured diversity and overall distributional agreement. IS was higher without truncation (2.514 versus 2.133) but is considered exploratory because it is based on an ImageNet classifier.

#### 3.2.2. Independent Pathologist Validation

Independent blinded validation supported improved image quality and subtype representation with per-class truncation. The independent pathologist rated per-class-truncated images higher than matched untruncated images (mean 2.31, bootstrap 95% CI 2.14–2.47 versus mean 1.51, 95% CI 1.29–1.74; Table 6). The paired mean difference was 0.80 (95% CI 0.57–1.03; paired permutation *p* < 0.00001), and 74.3% versus 52.9% of images were rated ≥2 (difference 21.4 percentage points, 95% CI 11.4–31.4). The original pathologist’s corresponding means were 2.11 (95% CI 2.01–2.20) and 1.91 (95% CI 1.73–2.10), respectively. Under the predefined tile-level scale (Figure 6), a score of 2 indicated that subtype-associated morphology was present despite image-quality limitations; 51 of 70 per-class-truncated images (72.9%) received scores ≥2 from the original pathologist. The mean of 2.11 therefore summarizes average tile-level subtype representation and image quality; it does not establish clinical validity, diagnostic usefulness, or the ability to diagnose a case from an isolated tile. Subtype-specific estimates are shown in Table 7; because only 10 images were evaluated per subtype and condition, these estimates have wide confidence intervals and are interpreted descriptively. Agreement across the 140 shared synthetic images was quantified by a quadratic-weighted κ of 0.599 (95% CI 0.489–0.691) and ICC (2,1) of 0.601 (95% CI 0.491–0.692); exact agreement was 52.1%, and 90.0% of ratings differed by no more than one category. In a secondary analysis grouping ratings as 0–1 versus 2–3, raw agreement was 80.7% (95% CI 75.0–86.4%) and binary κ was 0.571 (95% CI 0.444–0.691; Table S13). Thus, the pathologists agreed more often on whether subtype morphology was present than on the exact four-level grade, although 19.3% of ratings crossed this threshold. The independent pathologist rated the real reference tiles at a mean of 1.63 (95% CI 1.39–1.87), with 61.4% rated ≥2. These real tiles were sampled from the final training tile set and provide a reference for image quality and subtype representation among tiles used to train the generator. Comparisons with this curated reference set contextualize synthetic-image ratings but do not establish that synthetic images are superior to real tissue.

### 3.3. Exploratory Association Between Subtype Incidence and Mean Pathologist Rating

**Figure 8:**
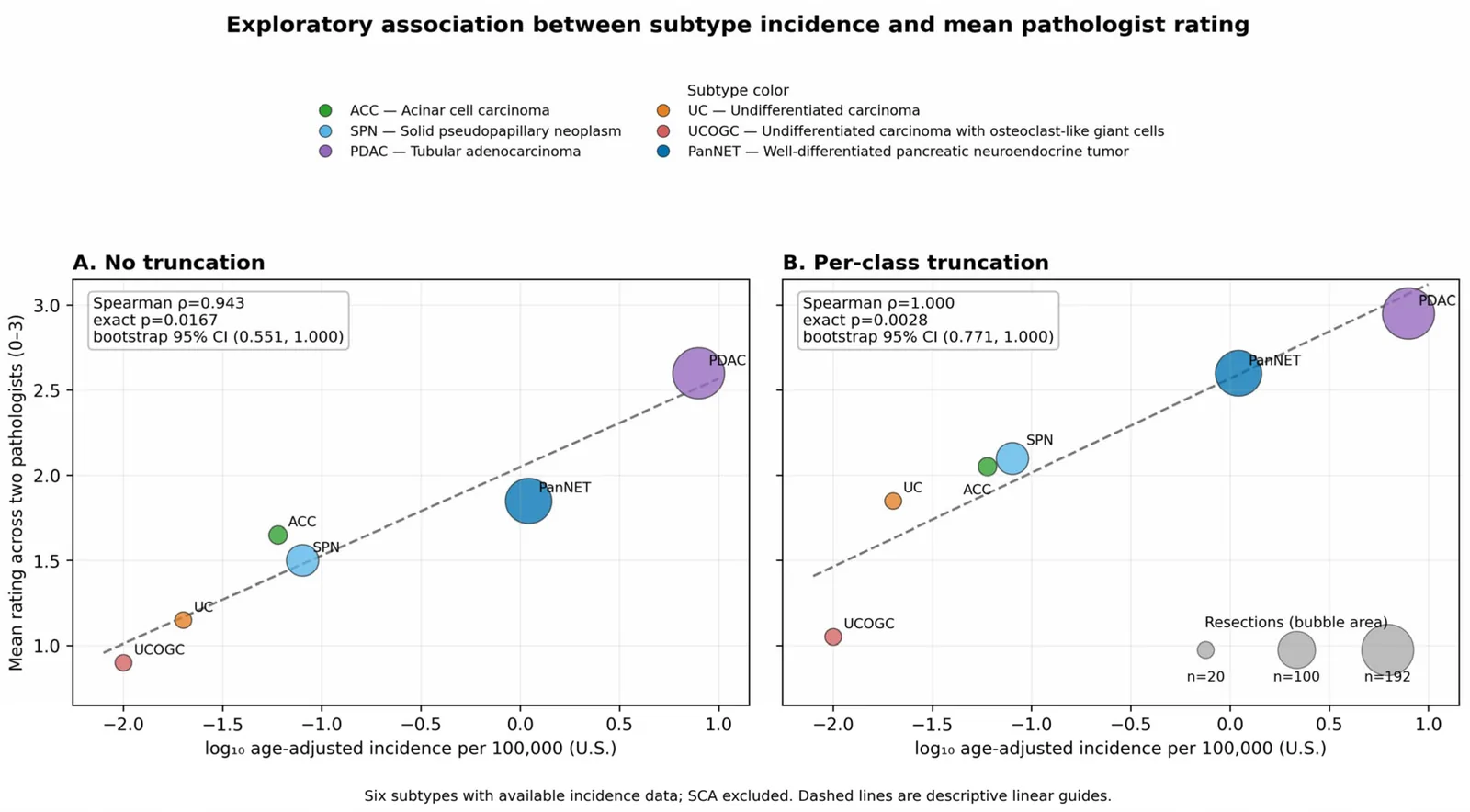
Exploratory association between age-adjusted U.S. incidence and mean synthetic-image rating across both pathologists for (**A**) no truncation and (**B**) per-class truncation. Each point represents one of six subtypes with available incidence data; bubble area is proportional to the number of resections in the training cohort.

Across the six subtypes with available incidence data, the mean rating across both pathologists showed a strong positive rank association with age-adjusted U.S. incidence under per-class truncation and no truncation, as shown in Figure 8. Microcystic serous cystadenoma was excluded because an incidence estimate was unavailable.

## 4. Discussion

In this work, we introduce a multi-step pipeline to construct a high-quality synthetic image generator for rare pancreatic neoplasms. We find that many of the fundamental principles that govern other realms of AI, such as class representation, apply here. By systematically eliminating image tiles containing background, technical artifacts, and biologically irrelevant or semi-imperfect regions, we iteratively constructed a high-quality training dataset of tiles containing diagnostically relevant morphology with a minimal annotation strategy. Below, we note some critical implications of our work.

A key implication is that subtype-conditional generative modeling in pathology is fundamentally data-centric. Generative models require a significantly higher tile-level data quality than WSI diagnosis models. This is because generative models fit the training distribution, meaning any noise—such as artifacts or non-tumor tiles—is likely to be reproduced in the synthetic output. In contrast, WSI diagnosis models [14,20] using a “bag-of-tiles” approach exhibit greater tolerance to tile-level noise. Consequently, we found that existing, common data preprocessing pipelines [14] were insufficient for the stringent quality requirements of generative modeling.

To address this, we leveraged open-source pathology models to compensate for the lack of large-scale annotations. We found certain tools, such as general tissue segmentation models, immediately useful for noise reduction, though the existing literature mainly focuses on coarse class classification, not rare tumor subtypes. Conversely, more specialized foundation models, such as the CONCH model, required adaptation to perform optimally on our specific, rare-subtype pancreatic tumor data, to ensure high quality of the training tiles. To fully exploit the foundation model, we curated an interpretable class-name lexicon through iterative refinement. By defining the lexicon through natural language descriptors of invariant tissue concepts (e.g., normal lymphocytes and chronic pancreatitis), we ensure its applicability across pancreatic datasets. This enables robust zero-shot performance with existing pathology foundation models on whole slide images of the pancreas. We further posit that, given access to high-quality tile-level annotations, this lexicon could be optimized automatically as a set of tunable hyperparameters.

Implementing these techniques requires an iterative approach with active pathologist involvement, as standard configurations often prove inadequate [50]. For instance, while we initially used a standard target tile size of 512 × 512, pathologist review revealed this field of view (0.256 mm × 0.256 mm) was insufficient for capturing the distinct morphology of subtypes such as solid pseudopapillary neoplasms. Based on this domain-specific feedback, we increased the input dimensions to 1024 × 1024 to ensure morphological integrity. Furthermore, the curation of an interpretable class-name lexicon benefited significantly from this human-in-the-loop approach. A salient example is observed in acinar cell carcinoma; despite its dense neoplastic cellularity, many tiles within a 1024 × 1024 field of view feature neoplastic cells but remain diagnostically indeterminate, as they do not exhibit features characteristic of acinar cell carcinoma. While not a complete resolution, integrating “morphologically indeterminate” and “non-diagnostic morphology” as keywords within the lexicon mitigated this issue, allowing the model to better handle ambiguous morphological patterns.

We posit that high-quality synthetic data generation in medicine is not just a modeling problem, but fundamentally a data curation and label fidelity problem requiring domain expertise and iterative refinement. We argue that this principle generalizes to other high-resolution medical and non-medical domains such as remote sensing, where gigapixel, heterogeneous data pose similar challenges [51]. However, specific components of the pipeline remain highly contextual. Elements such as the optimal prompt templates and interpretable class-name lexicons are domain-specific; therefore, applying this framework beyond H&E-stained WSIs of the pancreas requires re-specification and rigorous re-evaluation.

The quantitative distribution metrics and pathologist ratings captured complementary properties of truncation. Per-class truncation increased precision and density and improved independent pathologist ratings, but it also increased FID and KID and reduced recall and coverage. This pattern indicates concentration around a narrower set of prototypical subtype appearances rather than uniform improvement across all measures of image generation.

While pathologist input is essential to define diagnostic criteria and audit failure cases, the workflow avoids dense tile-level labeling by reserving expert time for targeted review. Independent blinded validation reduced the risk of optimization bias, and agreement between the original and independent pathologists was quantified. The weighted κ and ICC estimates indicate moderate agreement on the original four-level scale. After grouping ratings as 0–1 versus 2–3, the pathologists agreed on 80.7% of images, with binary κ = 0.571. This indicates that much of the disagreement concerned the exact quality grade rather than whether subtype morphology was present. The observed variability likely stems from evaluating isolated tiles rather than whole slides, which removes the broader context often crucial for challenging pancreatic interpretations. Additionally, because we did not evaluate the pathologists’ prior experience with tile-based assessments, differing familiarity levels may have also contributed to the disagreement.

In our cohort, higher-incidence pancreatic tumor subtypes tend to receive higher pathologist ratings. This association should be interpreted cautiously: while population incidence is a good proxy for representation, synthetic image quality may also reflect subtype-specific morphology and diagnostic complexity. However, the pattern is consistent with a broader concern in pathology AI: scale alone may not ensure balanced performance across rare organs, diseases, or morphologies [52,53]. If common morphologies dominate training data, foundation models may learn highly capable representations for prevalent entities while remaining less robust for rare or underrepresented subtypes. These findings motivate future studies to test whether targeted generative fine-tuning and expert-guided dataset enrichment using synthetic images can improve representation of rare pancreatic tumor morphologies and strengthen the diagnostic robustness of downstream pathology AI systems.

This work has several limitations. First, we developed and evaluated the workflow using a retrospective cohort from a single institution, so performance may not generalize to other institutions, scanner settings, staining protocols, or patient populations. Future work should compile and annotate multi-institutional cohorts that span scanners, staining protocols, and patient populations. Second, the human evaluation included two pathologists: one contributed to pipeline development and rated the synthetic images, while the second had no role in development and performed independent blinded ratings. Using an independent second pathologist reduced optimization bias, but two raters cannot characterize observer variability across the broader pathology community. Third, we evaluated only 10 synthetic images per subtype and condition, which increased uncertainty, especially for rare entities. Larger studies should assess more independently sampled images with multi-institutional panels that include pathologists experienced in rare pancreatic neoplasms. Fourth, we did not test whether synthetic-image exposure improves trainee learning or diagnostic performance. Future studies should conduct controlled educational studies that evaluate that outcome directly. Finally, our cohort comprised surgically resected neoplasms; future work should assess generalizability to smaller, differently processed EUS-guided biopsy specimens [54,55].

It is our hope that the approach presented here will aid in the creation of large numbers of synthetic images that encompass the entities pathologists-in-training should be familiar with, as well as the full spectrum of microscopic changes each of these entities can produce. Once created, such a database could serve as a foundation for uniform education in diagnostic pathology, and as an important augmentation of training data in large pathology AI models. Furthermore, if synthetic tiles can be shown to preserve diagnostic features without memorizing patient-specific information, they may also enable privacy-preserving sharing of rare subtypes to facilitate multi-center collaboration.

## 5. Conclusions

In conclusion, we developed an iterative, pathologist-in-the-loop workflow for generating clinical-grade synthetic pathology images across a diverse cohort of pancreatic tumors. By combining scalable tile pruning, prompt-optimized text-to-image retrieval, and pathologist-guided model refinement, the workflow produced synthetic images that achieved better-than-OK quality by blinded expert review. The results show that successful synthetic histology generation depends not only on generative model architecture, but also on careful data curation, label fidelity, and domain-specific oversight.

## Figures and Tables

**Table 1:** Number of resections and slides per primary diagnosis category after slide filtering, with a representative histology image for each disease.

Primary Diagnosis	Number of Resections	Number of Slides	Sample Tile
Tubular adenocarcinoma	192	276	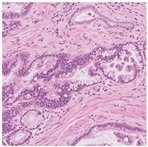
Well-differentiated pancreatic neuroendocrine tumor	153	254	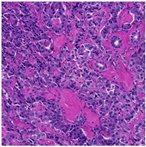
Solid pseudopapillary neoplasm	74	150	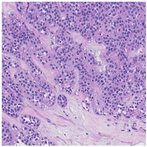
Microcystic serous cystadenoma	69	108	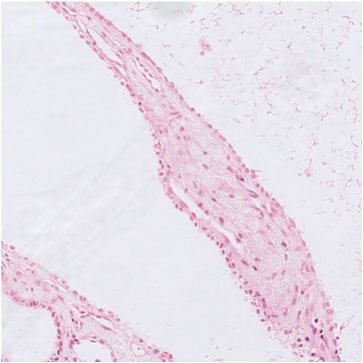
Acinar cell carcinoma	24	63	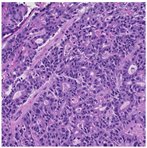
Undifferentiated carcinoma	20	25	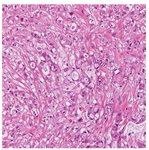
Undifferentiated carcinoma with osteoclast-like giant cells	20	56	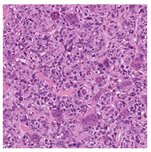

Sample histology images are 0.512 × 0.512 mm^2^.

**Table 2:** Comparison of estimated total manual annotation time for tile-level vs. slide-level annotations.

Annotation Level	Total Number of Slides	Total Number of Tiles	Approximate Time per Item (s)	Total Time (h)	Total Time (Days)
Tile level	932	5,137,856	5	7135.91	297.33
Slide-level	932	NA	80	20.71	0.86

**Table 3:** Comparison of baseline class names and our method.

	F1 Score	Recall	Precision
Baseline	0.55 (0.52, 0.58)	0.95 (0.93, 0.96)	0.46 (0.43, 0.50)
Ours	0.64 (0.61, 0.67)	0.74 (0.71, 0.76)	0.68 (0.65, 0.71)

**Table 4:** Comparison of baseline class names and our method for each subtype.

	F1		Recall		Precision	
	Baseline	Ours	Baseline	Ours	Baseline	Ours
Acinar cell carcinoma	0.63 (0.45, 0.78)	0.63 (0.55, 0.73)	0.94 (0.93, 0.96)	0.51 (0.43, 0.63)	0.54 (0.36, 0.70)	0.90 (0.83, 0.96)
Microcystic serous cystadenoma	0.60 (0.53, 0.66)	0.73 (0.67, 0.79)	0.98 (0.97, 0.99)	0.77 (0.73, 0.80)	0.48 (0.42, 0.54)	0.77 (0.70, 0.83)
Solid pseudopapillary neoplasm	0.82 (0.73, 0.90)	0.72 (0.60, 0.82)	0.86 (0.77, 0.93)	0.65 (0.54, 0.75)	0.81 (0.72, 0.89)	0.87 (0.77, 0.96)
Tubular adenocarcinoma	0.27 (0.24, 0.30)	0.39 (0.36, 0.43)	0.94 (0.92, 0.96)	0.70 (0.67, 0.74)	0.19 (0.16, 0.21)	0.35 (0.31, 0.39)
Undifferentiated carcinoma	0.55 (0.46, 0.65)	0.75 (0.67, 0.84)	0.91 (0.84, 0.97)	0.75 (0.66, 0.83)	0.44 (0.34, 0.55)	0.80 (0.71, 0.88)
Undifferentiated carcinoma with osteoclast-like giant cells	0.74 (0.69, 0.79)	0.75 (0.64, 0.83)	0.97 (0.96, 0.98)	0.74 (0.63, 0.83)	0.63 (0.57, 0.70)	0.82 (0.75, 0.87)
Well-differentiated pancreatic neuroendocrine tumor	0.61 (0.57, 0.66)	0.80 (0.77, 0.83)	0.98 (0.97, 0.99)	0.86 (0.84, 0.88)	0.50 (0.45, 0.55)	0.78 (0.75, 0.82)

**Table 5:** Quantitative evaluation metrics for synthetic images with per-class truncation and no truncation.

Condition	FID↓	KID↓	Precision↑	Recall↑	Density↑	Coverage↑	IS↑
ψ = 0.5	29.084	0.01678 ± 0.00116	0.743 ± 0.003	0.104 ± 0.002	1.348 ± 0.012	0.606 ± 0.002	2.133 ± 0.076
ψ = 1.0	6.637	0.00328 ± 0.00063	0.631 ± 0.004	0.460 ± 0.003	0.775 ± 0.005	0.781 ± 0.003	2.514 ± 0.085

FID is a single estimate against the entire real tiles. KID, precision, recall, density, coverage and IS are mean ± standard deviation.

**Table 6:** Overall pathologist ratings by source and truncation condition.

Source/Condition	Rater	n	Mean (95% CI)	Median [IQR]	≥2
Per-class truncation	Original	70	2.11 (2.01, 2.20)	2.00 [1.00, 3.00]	51/70 (72.9%)
Per-class truncation	Independent	70	2.31 (2.14, 2.47)	3.00 [1.25, 3.00]	52/70 (74.3%)
No truncation	Original	70	1.91 (1.73, 2.10)	2.00 [1.00, 3.00]	45/70 (64.3%)
No truncation	Independent	70	1.51 (1.29, 1.74)	2.00 [1.00, 2.00]	37/70 (52.9%)
Real reference	Independent	70	1.63 (1.39, 1.87)	2.00 [1.00, 2.00]	43/70 (61.4%)

Mean-score 95% CIs are subtype-stratified bootstrap intervals; ≥2 indicates that subtype morphology was present.

**Table 7:** Subtype-specific ratings from the independent pathologist.

Subtype	Per-Class	No Truncation	Real Reference	Paired Δ (95% CI)
Acinar cell carcinoma	1.90 (1.40, 2.40)	1.50 (0.80, 2.20)	1.40 (0.70, 2.10)	0.40 (−0.30–1.20)
Microcystic serous cystadenoma	2.90 (2.70, 3.00)	2.00 (1.60, 2.40)	1.40 (0.90, 1.80)	0.90 (0.50–1.30)
Solid pseudopapillary neoplasm	2.20 (1.60, 2.80)	1.30 (0.50, 2.10)	1.10 (0.50, 1.70)	0.90 (0.20–1.70)
Tubular adenocarcinoma	2.90 (2.70, 3.00)	2.30 (2.00, 2.60)	2.10 (1.40, 2.70)	0.60 (0.30–0.90)
Undifferentiated carcinoma	2.40 (1.90, 2.80)	1.20 (0.40, 2.00)	1.70 (1.00, 2.40)	1.20 (0.50–2.00)
Undifferentiated carcinoma with osteoclast-like giant cells	1.20 (1.00, 1.60)	0.90 (0.70, 1.00)	1.90 (1.20, 2.60)	0.30 (0.00–0.70)
Well-differentiated pancreatic neuroendocrine tumor	2.70 (2.30, 3.00)	1.40 (0.60, 2.10)	1.80 (1.30, 2.20)	1.30 (0.60–2.10)

Values are mean (bootstrap 95% CI); Δ is per-class truncation minus no truncation. Each source/condition contains 10 images per subtype.

## Data Availability

High-resolution image tiles used for generative model training, as well as our cohort of generated histology tiles, are available from the corresponding author upon request.

## Supplementary Materials

The following supporting information can be downloaded at https://www.mdpi.com/article/10.3390/cancers18183004/s1, Figure S1. Development-set F1 across candidate utility-score thresholds for each case-disjoint outer fold; Figure S2. Our prompt to generate prompt templates; Figure S3. Example synthetic images generated by the model using random sampling; Figure S4. Example synthetic images generated by the model using oversampling by case ID; Figure S5. Example synthetic images generated by the model using two-stage stratified sampling; Figure S6. Synthetic tubular adenocarcinoma image with per-class truncation (left) and no truncation (right); Figure S7. Synthetic microcystic serous cystadenoma image with per-class truncation (left) and no truncation (right); Figure S8. Ten synthetic images without truncation for each subtype; Figure S9. Ten synthetic images with per-class truncation for each subtype; Figure S10. Highest-CONCH-similarity synthetic/training pair for each subtype and truncation condition; Table S1. Case-disjoint utility-score threshold selection and held-out performance; Table S2. Comparison of different similarity score threshold values; Table S3. Complete candidate prompt templates, listed in prompt-ID order from 0 to 48; Table S4. Case-disjoint cross-validation of prompt-template selection; Table S5. Our list of alternative class names; Table S6. Primary diagnosis synonyms; Table S7. Excluded non-target class terms used as negative filters for certain primary diagnosis categories; Table S8. Comparison of minimum FID and final epoch FID values for models trained with random sampling; Table S9. The last epoch FID and minimum FID versus the hyperparameter; Table S10. Quantitative evaluation metrics for synthetic images; Table S11. The 2020 age-adjusted incidence per 100,000 in the U.S. for the selected seven subtypes. Table S12. Comparison of truncation strategies by the original pathologist. Table S13. Inter-rater agreement on shared synthetic images; Table S14. Secondary binary agreement after grouping ratings 0–1 (subtype morphology not established) versus 2–3 (subtype morphology present); Table S15. Full subtype-specific rating distributions; Table S16. CONCH top-1 training-tile similarity distributions for saved synthetic images; Table S17. Case-level F1 and one-vs-rest AUROC for real-only and synthetic-augmented classifiers on the independent same-institution test cohort [56,57,58].

## Abbreviations

The following abbreviations are used in this manuscript:

AI	Artificial intelligence
FID	Fréchet Inception Distance
GANs	Generative adversarial networks
GPU	Graphics processing unit
H&E	Hematoxylin and eosin
TLA	Three-letter acronym
LD	Linear dichroism
