# Supplementary material for: An Iterative, Pathologist-in-the-Loop Workflow for Generation of Clinical-Grade Synthetic Pathology Images in a Diverse Cohort of Pancreatic Tumors
Source: Cancers (Basel). 2026 Sep 16;18(18):3004. doi: 10.3390/cancers18183004 (PMC13605902; doi:10.3390/cancers18183004)
Supplement: Supplementary file 1 [file cancers-18-03004-s001.zip › cancers-4480343-supplementary.pdf]

Supplementary Material

Section 1. Threshold optimization for tile utility scores

To evaluate utility-score threshold selection without case leakage, we used 395 professionally annotated tiles (282 high-quality and 113 low-quality) from eight coded cases. We assigned cases, rather than tiles, to five outer folds. For each outer fold, we pooled the other four folds, evaluated thresholds from 0.00 to 1.00 in increments of 0.01, and selected the lowest threshold attaining the maximum development-set F1. We then applied this fixed threshold to the untouched outer fold. All 95% confidence intervals were estimated with 10,000 hierarchical bootstrap replicates that resampled cases and then tiles within each sampled case (Table S1 and Figure S1).

Table S1. Case-disjoint utility-score threshold selection and held-out performance.

Outer fold	Selected threshold	Development F1	Held-out F1 (95% CI)
1	0.19	0.880	0.943 (0.843, 0.990)
2	0.19	0.899	0.889 (0.750, 0.974)
3	0.19	0.909	0.846 (0.083, 1.000)
4	0.29	0.895	0.775 (0.667, 0.871)
5	0.19	0.911	0.789 (0.676, 0.881)
Mean (SD)	0.210 (0.045)	0.899 (0.012)	0.848 (0.070)
Pooled	N/A	N/A	0.866 (0.750, 0.943)

For final preprocessing, we retained the utility-score cutoff of 0.22 obtained after fitting the threshold on all 395 annotations. This full-data cutoff was used to curate the generative model training set. Because the annotation sample contained only eight cases, each outer fold included one or two held-out cases. Confidence intervals for one-case folds are conditional within-case tile-sampling intervals and do not estimate between-case variability. The pooled case-aware interval is therefore the primary uncertainty estimate.

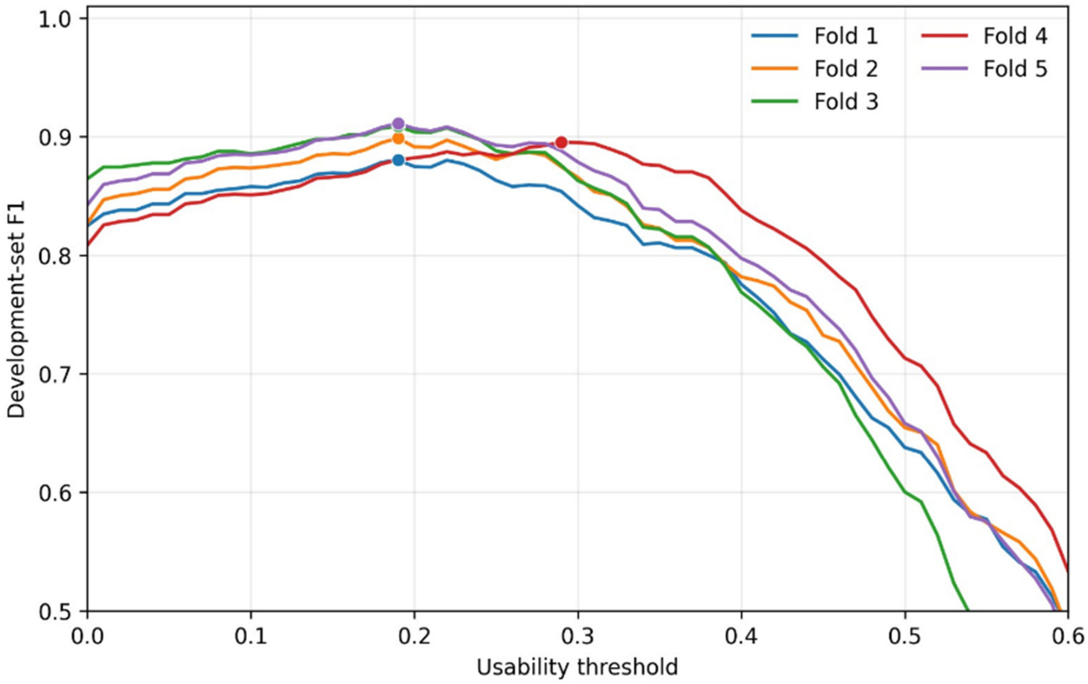

Figure S1. Development-set F1 across candidate utility-score thresholds for each case-disjoint outer fold. Points mark the selected threshold.

Section 2. Text-to-image retrieval ablation study

We performed an ablation study to assess the impact of the similarity score threshold on retrieval performance. This threshold (default = 0.01) is used to assign a tile to an 'other' category if its similarity scores for all classes fall below this value, a mechanism designed to reduce false positives from low-confidence classifications. The choice of similarity score threshold had a minimal impact on retrieval performance (F1-score, precision, and recall), as shown in Supplementary Table 2. The observed insensitivity to this threshold is likely due to our comprehensive class name lexicon, which covers most non-tumor or non-cyst categories and thus provides a high-confidence match for most tiles.

Table S2. Comparison of different similarity score threshold values.

Similarity Score Threshold	F1 (95% CI)	Precision (95% CI)	Recall (95% CI)
0.00	0.64 (0.62, 0.66)	0.68 (0.66, 0.70)	0.74 (0.72, 0.75)
0.01	0.64 (0.62, 0.66)	0.68 (0.66, 0.70)	0.74 (0.72, 0.75)
0.02	0.64 (0.62, 0.66)	0.68 (0.66, 0.70)	0.74 (0.72, 0.75)
0.03	0.64 (0.62, 0.66)	0.68 (0.66, 0.70)	0.74 (0.72, 0.75)
0.04	0.64 (0.62, 0.66)	0.68 (0.66, 0.70)	0.74 (0.72, 0.75)
0.05	0.64 (0.62, 0.66)	0.68 (0.66, 0.70)	0.74 (0.72, 0.75)
0.06	0.64 (0.62, 0.66)	0.68 (0.66, 0.70)	0.73 (0.72, 0.75)
0.07	0.64 (0.62, 0.66)	0.68 (0.66, 0.70)	0.73 (0.72, 0.75)
0.08	0.64 (0.62, 0.66)	0.68 (0.66, 0.70)	0.73 (0.72, 0.75)
0.09	0.64 (0.62, 0.66)	0.68 (0.66, 0.70)	0.73 (0.72, 0.75)
0.10	0.64 (0.62, 0.66)	0.68 (0.66, 0.70)	0.73 (0.72, 0.75)

To prospectively assess the robustness of prompt-template selection, we evaluated the 49 candidate templates listed in Table S3 using case-disjoint five-fold cross-validation. Stratified grouped splitting approximately balanced the slide-level primary-diagnosis distribution while assigning every slide from a coded case to one outer fold. For each outer fold, we selected the prompt with the highest mean slide-level F1 across the other four development folds, resolving an exact tie in favor of the lower prompt ID, and then evaluated that fixed prompt on the untouched held-out cases. 95% confidence intervals used 10,000 case-cluster bootstrap replicates.

Table S3. Complete candidate prompt templates, listed in prompt-ID order from 0 to 48.

Prompt template
this is a photomicrograph illustrating {text}, H&E stain
this is {text}, H&E stain
this is a histology image, {text}, H&E stain
this is {text}, pancreas, H&E stain
this is a histology image of pancreas, {text}, H&E stain
this is a photomicrograph of {text}, pancreas, H&E stain
this is a photomicrograph illustrating {text} in pancreas, H&E stain
this is an H&E stained histology image of pancreas showing {text}
this is a histology image of {text}, H&E stain
this is a histology image of {text} in pancreas, H&E stain
this is a histopathology image of {text} in pancreas, H&E stain
this is {text} H&E stain
this is an image showing {text}, H&E stain
a photomicrograph showing {text}, H&E stain
a photomicrograph illustrating {text}, pancreas, H&E stain
a histopathology image of {text}, H&E stain
a histopathology slide showing {text}, H&E stain
a histopathological slide of {text}, H&E stain
a histology image of {text}, H&E stained
a histology image of pancreas illustrating {text}, H&E stain
an H&E stained slide showing {text}, pancreas
an H&E stained image of {text}, pancreas

an H&E image of pancreas showing {text}
an H&E stained photograph of {text}, pancreas
this is a histopathological image of {text} in pancreas
this is a histopathology slide showing {text}
this is a photomicrograph of {text} in pancreas
this is a histology photograph showing {text}, H&E stain
a histopathological photograph showing {text}, H&E stain
a histology slide of {text}, H&E stain
a histology photograph of {text}, H&E stained
an image of {text}, pancreas, H&E stain
an H&E stained photomicrograph illustrating {text} in pancreas
an H&E stained histology image showing {text}, pancreas
a histopathological image showing {text} in pancreas
a histopathology slide illustrating {text}, H&E stain
presence of {text} in pancreas, H&E stain
an H&E stained photomicrograph of {text}
an H&E stained histology slide of pancreas showing {text}
an H&E stained histology photograph illustrating {text} in pancreas
an H&E stained histopathology slide showing {text}
a histology image of pancreas showing the presence of {text}
a histopathological slide illustrating {text} in pancreas
a histology image showing {text}, pancreas, H&E stain
this is a histology photograph illustrating {text} in pancreas
a photomicrograph of pancreas with {text}, H&E stain
an example of {text} in pancreas, H&E stain
there is {text}, H&E stain
shows {text} in pancreas, H&E stain

Prompt 19 was selected independently in all five outer folds. The pooled out-of-fold mean slide-level F1 was 0.650 (95% CI, 0.620–0.678; Table S4). This analysis demonstrates stable internal case-disjoint prompt selection, but it is not external independent-dataset validation.

The final preprocessing pipeline used prompt 45, "a photomicrograph of pancreas with {text}, H&E stain," which was selected during the initial-lexicon stage to optimize the entire cohort. It achieved a pooled F1 score of 0.64, recall of 0.74 and precision of 0.68, performing comparably to prompt 19.

Table S4. Case-disjoint cross-validation of prompt-template selection. All five folds selected prompt 19: "a histology image of pancreas illustrating {text}, H&E stain." Values are mean slide-level F1; confidence intervals are percentile 95% CIs from 10,000 case-cluster bootstrap replicates.

Outer fold	Selected template	Development mean F1	Held-out mean F1 (95% CI)
1	19	0.647	0.661 (0.573, 0.734)
2	19	0.646	0.667 (0.614, 0.716)
3	19	0.647	0.660 (0.606, 0.713)
4	19	0.659	0.616 (0.557, 0.673)
5	19	0.651	0.646 (0.580, 0.710)
Pooled	19	N/A	0.650 (0.620, 0.678)

All five folds selected prompt 19: "a histology image of pancreas illustrating {text}, H&E stain." Values are mean slide-level F1; confidence intervals are percentile 95% CIs from 10,000 case-cluster bootstrap replicates.

Section 3. Class lexicon

Table S5. Our list of alternative class names.

**Category**	**Class names**
Secondary diagnosis	Include all the secondary diagnosis names if available.
normal	normal
inflammation	chronic pancreatitis
	fibroinflammatory pancreatitis
	macrophage
cyst	retention cyst
	mucinous cyst
fat	adipose
	adipose tissue
	adipocytes
	fat
	fat cells
normal stroma	normal stroma
	normal stromal cells
	normal stromal tissue
	normal fibroblast
	normal myofibroblast
background and artifacts	background
	Pen marking
	empty space
	background artifacts
	poor quality
	blur
	air bubbles
	poor staining
	tissue folding
	out-of-focus
	formalin pigment
benign pancreatic structures	normal islets of Langerhans
	normal islets
	normal duct
	smooth muscle
	normal acini
	normal acinus
	normal acinar cell
	normal nerve
	normal nerve fiber
	normal pancreatic stellate cell
	normal lymphocyte
	normal islet cell
	normal ductal cell
	normal ductal epithelial cell
indeterminate	morphologically indeterminate
	non-diagnostic morphology

Table S6. Primary diagnosis synonyms.

Primary diagnosis	Primary diagnosis synonyms
tubular adenocarcinoma	ductal adenocarcinoma
undifferentiated carcinoma	anaplastic carcinoma
undifferentiated carcinoma with osteoclast-like giant cells	anaplastic carcinoma with osteoclast-like giant cells<br>pleomorphic carcinoma with osteoclast-like giant cells<br>undifferentiated carcinoma with osteoclastic giant cells<br>undifferentiated pancreatic carcinoma with osteoclastic giant cells

Table S7. Excluded non-target class terms used as negative filters for certain primary diagnosis categories.

Primary diagnosis	Excluded non-target class terms
undifferentiated carcinoma	moderately differentiated carcinoma
undifferentiated carcinoma with osteoclast-like giant cells	Undifferentiated carcinoma<br>anaplastic carcinoma<br>pleomorphic carcinoma<br>undifferentiated pancreatic carcinoma<br>pleomorphic large cell carcinoma<br>pleomorphic giant cell carcinoma<br>spindle cell carcinoma<br>sarcomatoid carcinoma
microcystic serous cystadenoma	pseudocyst

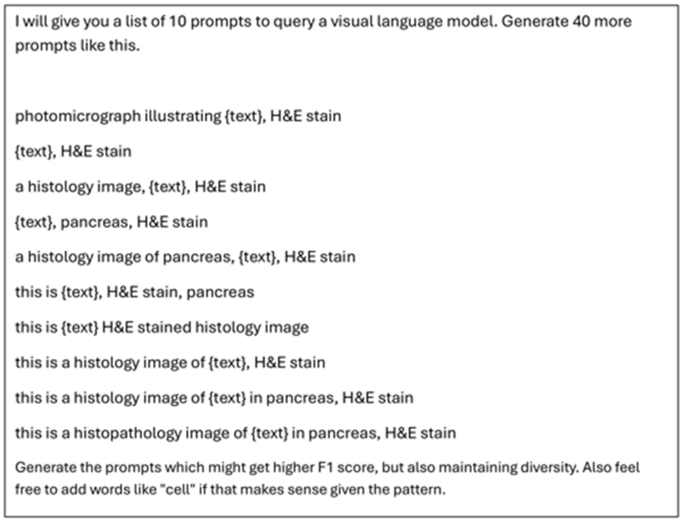
I will give you a list of 10 prompts to query a visual language model. Generate 40 more prompts like this.

photomicrograph illustrating {text}, H&E stain

{text}, H&E stain

a histology image, {text}, H&E stain

{text}, pancreas, H&E stain

a histology image of pancreas, {text}, H&E stain

this is {text}, H&E stain, pancreas

this is {text} H&E stained histology image

this is a histology image of {text}, H&E stain

this is a histology image of {text} in pancreas, H&E stain

this is a histopathology image of {text} in pancreas, H&E stain

Generate the prompts which might get higher F1 score, but also maintaining diversity. Also feel free to add words like "cell" if that makes sense given the pattern.

Figure S2. Our prompt to generate prompt templates.

Section 4. ID oversampling and R1 regularization impact model fidelity

In our database, each identity corresponds to a unique case (one surgically resected neoplasm). A case may include multiple whole slide images, and we cropped each slide into tens of thousands of tiles. The primary diagnosis served as the case label. We explored three sampling strategies: First, we applied random sampling. Second, we oversampled by case ID to ensure equal representation for each case, preventing cases with numerous patches from dominating training. Third, we implemented a two-stage stratified sampling approach by both label category and case ID, guaranteeing equal representation of each label and each case within every label.

We conducted an ablation study with these three sampling methods, training each model on 3,000,000 images. For evaluation, we used the Fréchet Inception Distance (FID) to quantify the similarity between distributions of synthetic images and the training set under each configuration. Table S8 presents both the minimum FID and the FID at the final epoch for each sampling configuration.

Table S8. Comparison of minimum FID and final epoch FID values for models trained with random sampling, oversampling by case ID, and two-stage stratified sampling methods.

Sampling Method	FID	FIDmin
Random sampling	7.83	7.83
Oversample by case ID	**5.36**	**5.02**
Two-stage stratified sampling	8.38	8.27

In Figures S3-S5, we present five representative synthetic images for each of four selected diagnostic categories. Using random sampling, the model consistently demonstrates severe mode collapse across all categories, with most generated images exhibiting a repetitive rounded morphology. This likely occurs because a small number of cases with the highest tile counts dominate the training data. With two-stage stratified sampling, we observe that, although pattern repetition is absent across categories, the microcystic serous cystadenoma and tubular adenocarcinoma group display mode collapse, as all synthetic images within this category appear highly similar. This phenomenon likely results from excessive duplication of certain samples during two-stage stratified sampling and, consequently, mode collapse. In contrast, oversampling by case ID produces the most diverse set of images within each diagnostic category, yielding superior results in terms of visual heterogeneity within the same training time.

In addition, we conducted an ablation study to assess the impact of R1 regularization weight γ values on model performance. Following established guidelines [24], we tested four different values in the range $[\gamma_0/5, \gamma_0 \times 5]$, where γ_0=0.002× (image width×image height)/batch size. Under each setting, we trained the model for 3,000,000 images. In Table S9, we compared the final and minimum FID values, which confirmed that the γ parameter significantly impacts the final model quality. We used the optimal γ=33 to train our final model.

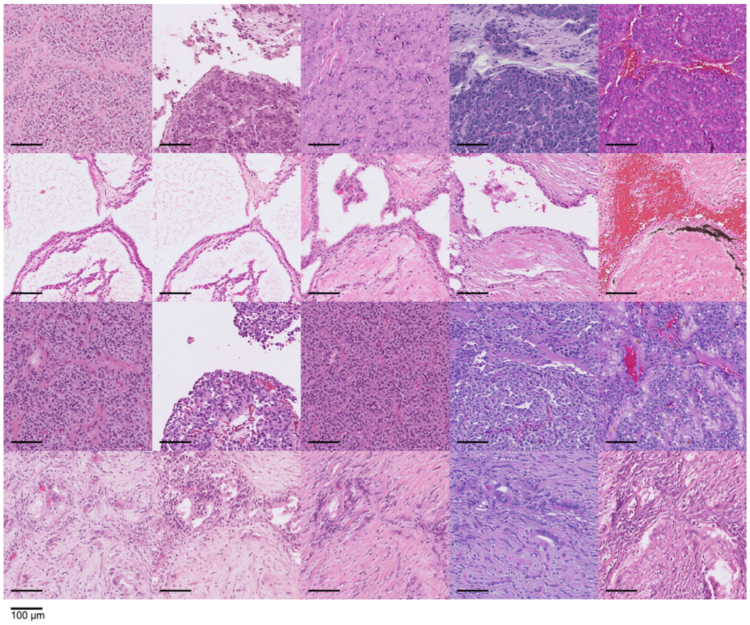

Figure S3. Example synthetic images generated by the model using random sampling. The first through fourth rows correspond to acinar cell carcinoma, microcystic serous cystadenoma, solid pseudopapillary neoplasm, and tubular adenocarcinoma, respectively.

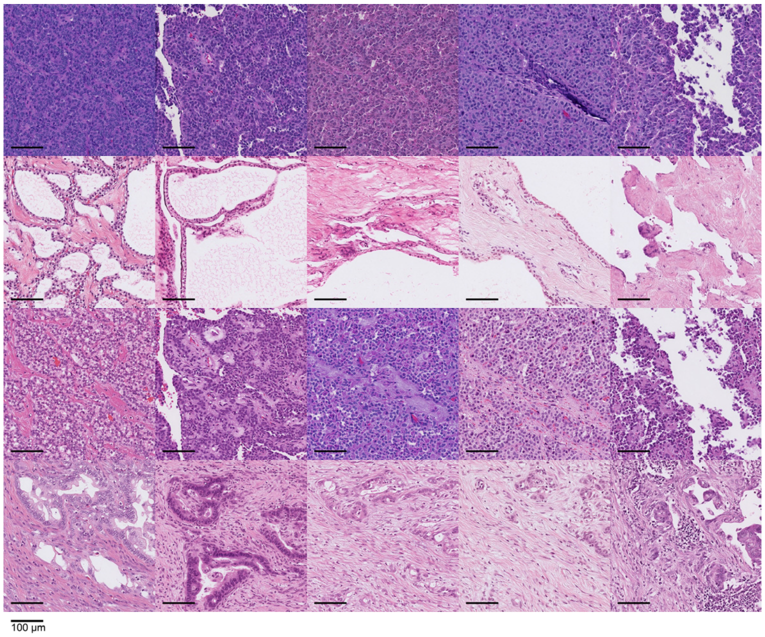

Figure S4. Example synthetic images generated by the model using oversampling by case ID. The first through fourth rows correspond to acinar cell carcinoma, microcystic serous cystadenoma, solid pseudopapillary neoplasm, and tubular adenocarcinoma, respectively.

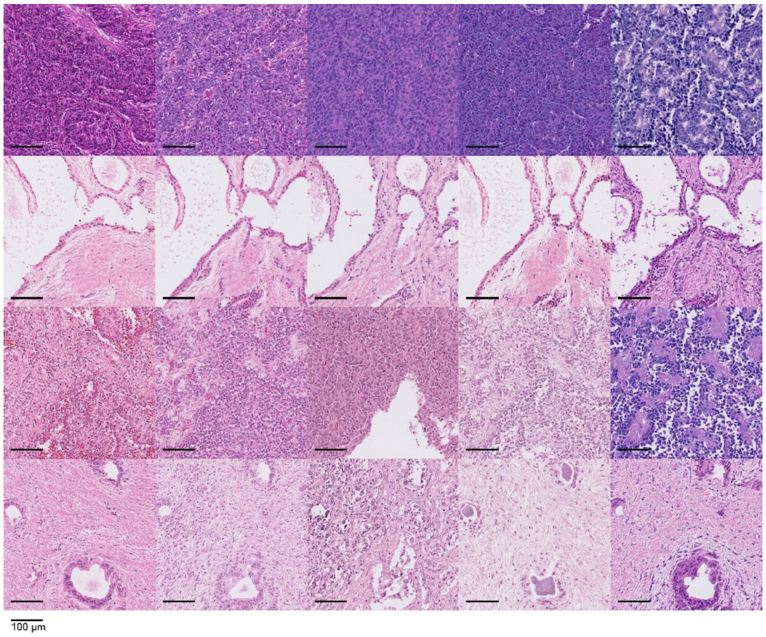

Figure S5. Example synthetic images generated by the model using two-stage stratified sampling. The first through fourth rows correspond to acinar cell carcinoma, microcystic serous cystadenoma, solid pseudopapillary neoplasm, and tubular adenocarcinoma, respectively.

Table S9. The last epoch FID and minimum FID versus the hyperparameter. .

γ	FID	FIDmin
8	7.85	7.85
16	8.81	8.65
33	**5.36**	**5.02**
66	5.62	5.53

Section 5. Post-processing truncation

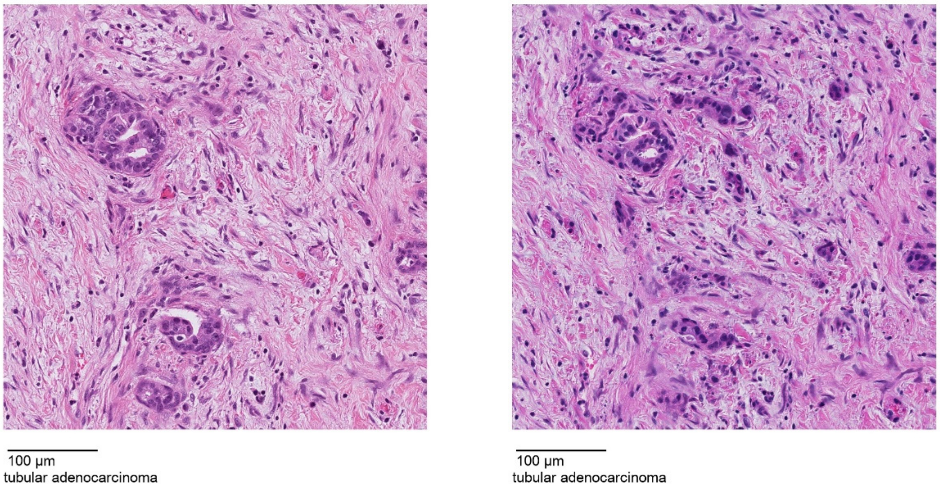

Figure S6. Synthetic tubular adenocarcinoma image with per-class truncation (left) and no truncation (right). Corresponding expert ratings were “very good” and “ok” respectively.

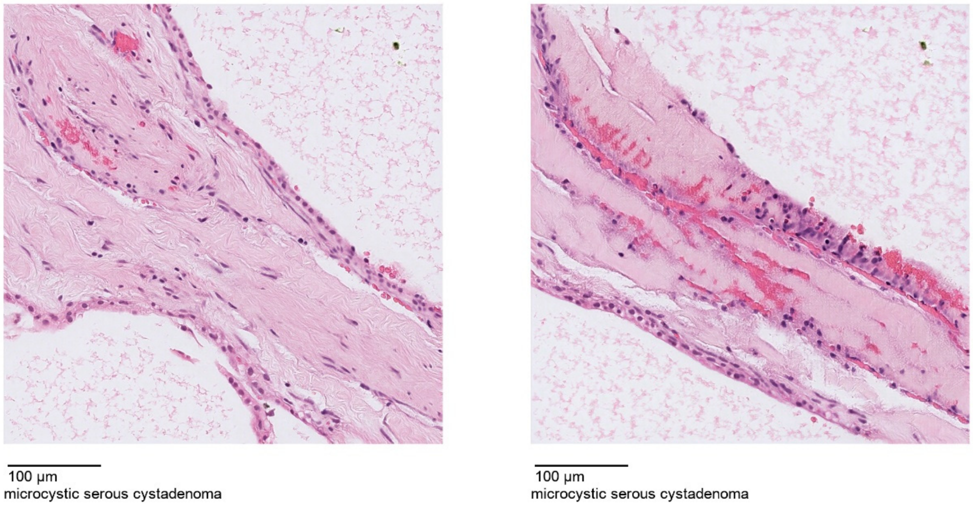

Figure S7. Synthetic microcystic serous cystadenoma image with per-class truncation (left) and no truncation (right). Corresponding expert ratings were “very good” and “ok” respectively.

Table S10 reports the subtype-specific quantitative evaluation metrics for no truncation and per-class truncation. These objective feature-space measures complement, but do not replace, the pathologist assessment of tile-level image quality and subtype representation.

Table S10. Quantitative evaluation metrics for synthetic images with per-class truncation and no truncation for each subtype. .

Subtype	Condition	FID↓	KID↓	Precision↑	Recall↑	Density↑	Coverage↑	IS↑
acinar cell carcinoma	ψ = 0.5	44.778	0.02678 ± 0.00113	0.638 ± 0.022	0.086 ± 0.003	0.855 ± 0.037	0.459 ± 0.006	1.991 ± 0.069
acinar cell carcinoma	ψ = 1.0	19.268	0.00657 ± 0.00087	0.497 ± 0.005	0.352 ± 0.004	0.570 ± 0.014	0.590 ± 0.004	2.348 ± 0.100
microcystic serous cystadenoma	ψ = 0.5	52.720	0.03538 ± 0.00123	0.797 ± 0.024	0.058 ± 0.004	2.289 ± 0.015	0.682 ± 0.008	2.118 ± 0.055

microcystic serous cystadenoma	ψ = 1.0	18.784	0.01108 ± 0.00106	0.694 ± 0.011	0.310 ± 0.006	1.101 ± 0.013	0.827 ± 0.007	2.788 ± 0.074
solid pseudopapillary neoplasm	ψ = 0.5	42.144	0.03141 ± 0.00150	0.841 ± 0.008	0.089 ± 0.004	1.159 ± 0.015	0.609 ± 0.003	1.847 ± 0.056
solid pseudopapillary neoplasm	ψ = 1.0	13.136	0.00555 ± 0.00060	0.627 ± 0.010	0.460 ± 0.007	0.637 ± 0.008	0.796 ± 0.012	2.532 ± 0.108
tubular adenocarcinoma	ψ = 0.5	52.144	0.04051 ± 0.00134	0.695 ± 0.019	0.066 ± 0.003	1.863 ± 0.060	0.576 ± 0.008	1.644 ± 0.038
tubular adenocarcinoma	ψ = 1.0	13.094	0.00576 ± 0.00057	0.658 ± 0.004	0.409 ± 0.010	0.948 ± 0.017	0.832 ± 0.004	2.187 ± 0.055
undifferentiated carcinoma	ψ = 0.5	65.141	0.05302 ± 0.00213	0.660 ± 0.023	0.045 ± 0.002	1.032 ± 0.021	0.393 ± 0.006	1.414 ± 0.034
undifferentiated carcinoma	ψ = 1.0	19.169	0.00973 ± 0.00093	0.626 ± 0.021	0.371 ± 0.007	0.662 ± 0.010	0.645 ± 0.007	1.702 ± 0.057
undifferentiated carcinoma with osteoclast-like giant cells	ψ = 0.5	66.834	0.06768 ± 0.00293	0.712 ± 0.025	0.056 ± 0.004	0.690 ± 0.044	0.412 ± 0.009	1.157 ± 0.025
undifferentiated carcinoma with osteoclast-like giant cells	ψ = 1.0	18.678	0.01030 ± 0.00098	0.603 ± 0.011	0.415 ± 0.006	0.586 ± 0.018	0.623 ± 0.013	1.644 ± 0.050
well-differentiated pancreatic neuroendocrine tumor	ψ = 0.5	37.619	0.02605 ± 0.00145	0.800 ± 0.018	0.100 ± 0.004	1.361 ± 0.028	0.685 ± 0.003	1.723 ± 0.044
well-differentiated pancreatic neuroendocrine tumor	ψ = 1.0	9.528	0.00292 ± 0.00037	0.643 ± 0.006	0.528 ± 0.003	0.775 ± 0.010	0.852 ± 0.003	2.420 ± 0.093

FID is a single estimate against the entire real tiles. KID, precision, recall, density, coverage and IS are mean ± standard deviation.

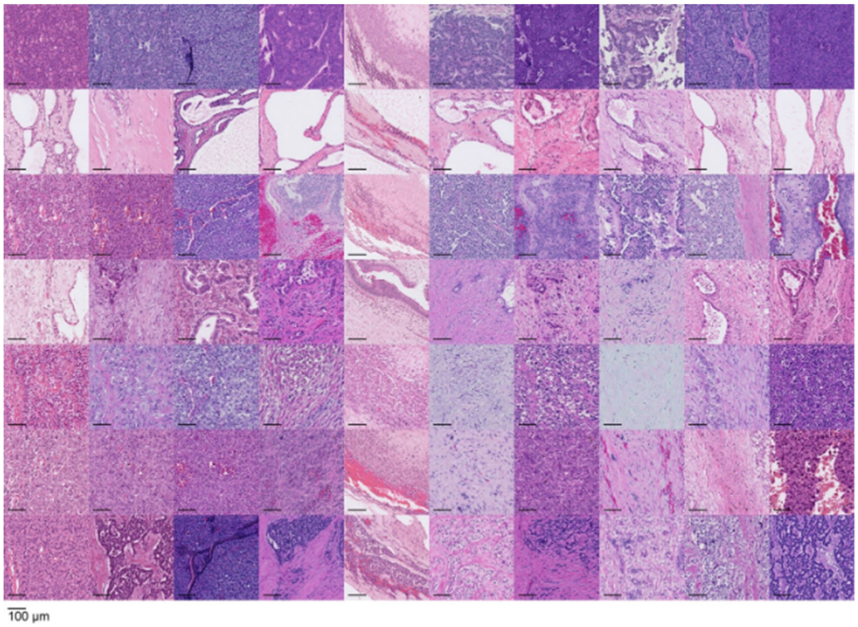

Figure S8. Ten synthetic images without truncation for each subtype. From top to bottom, rows show acinar cell carcinoma, microcystic serous cystadenoma, solid pseudopapillary neoplasm, tubular adenocarcinoma, undifferentiated carcinoma, undifferentiated carcinoma with osteoclast-like giant cells, and well-differentiated pancreatic neuroendocrine tumor.

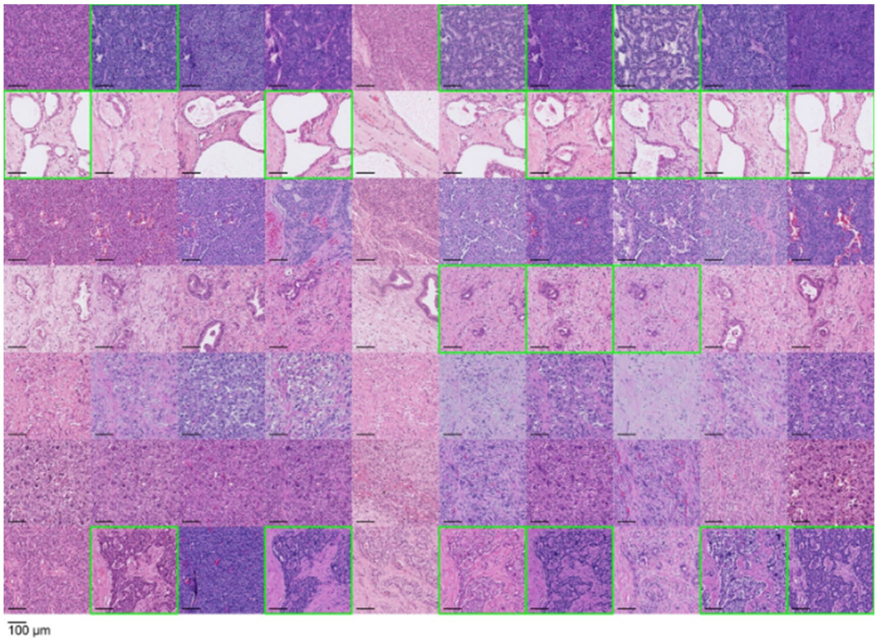

Figure S9. Ten synthetic images with per-class truncation for each subtype, shown in the same row order as Figure S8. Green boxes highlight visually similar patterns within subtype rows.

Section 6. The 2020 age-adjusted incidence rates

Table S11 provides the 2020 age-adjusted incidence rates for the selected seven subtypes. The incidence rates for tubular adenocarcinoma and well-differentiated pancreatic neuroendocrine tumor were reported in a previous study [56]. The incidence rates for the remaining subtypes were calculated using SEER*Stat software (version 9.0.42.2). The incidence rate for microcystic serous cystadenoma is not available in the SEER research data.

Table S11. The 2020 age-adjusted incidence per 100,000 in the U.S. for the selected seven subtypes.

Primary diagnosis	2020 Age-adjusted incidence per 100,000 in the U.S.
Acinar cell carcinoma	0.06
Microcystic serous cystadenoma	Not available
Solid pseudopapillary neoplasm	0.08
Tubular adenocarcinoma	7.92
Undifferentiated carcinoma	0.02
Undifferentiated carcinoma with osteoclast-like giant cells	0.01
Well-differentiated pancreatic neuroendocrine tumor	1.10

Section 7. Extended Pathologist-Rating Analyses

The independent pathologist rated the same 140 synthetic images as the original pathologist and an additional 70 real reference tiles. The detailed agreement and per-subtype results are reported below.

Based on Table S12, we can compare the no truncation, per-class truncation, and class-agnostic truncation. Per-class truncation yields the highest mean rating by the original pathologist. But we should also note that this improvement in fidelity represents a trade-off with diversity, as shown in Figures S8 and S9.

Table S12. Comparison of truncation strategies by the original pathologist. The class-agnostic condition was not evaluated by the independent pathologist. Confidence intervals are subtype-stratified bootstrap 95% CIs.

Truncation strategy	*n*	Mean (SD)	Bootstrap 95% CI	≥2
Per-class truncation	70	2.11 (0.84)	2.01–2.20	51/70 (72.9%)
Class-agnostic truncation	70	1.97 (1.01)	1.84–2.10	44/70 (62.9%)
No truncation	70	1.91 (1.05)	1.73–2.10	45/70 (64.3%)

Table S13. Inter-rater agreement on shared synthetic images. .

Condition	*n*	Weighted κ (95% CI)	ICC(2,1) (95% CI)	Exact	Within 1	Bias
Overall	140	0.599 (0.489–0.691)	0.601 (0.491–0.692)	52.1%	90.0%	-0.10
no_trunc	70	0.591 (0.436–0.716)	0.594 (0.440–0.719)	44.3%	88.6%	-0.40
per_class	70	0.570 (0.426–0.700)	0.574 (0.430–0.703)	60.0%	91.4%	0.20

Confidence intervals are subtype- and condition-stratified bootstrap 95% CIs; bias is the independent minus original mean rating.

Table S14. Secondary binary agreement after grouping ratings 0–1 (subtype morphology not established) versus 2–3 (subtype morphology present). .

Condition	*n*	Raw agreement (95% CI)	Binary κ (95% CI)	Positive agreement	Negative agreement
Overall	140	80.7% (75.0–86.4)	0.571 (0.444–0.691)	85.4% (80.4–89.9)	71.6% (62.4–79.6)
No truncation	70	80.0% (71.4–88.6)	0.593 (0.424–0.759)	82.9% (74.6–90.2)	75.9% (65.2–85.7)
Per-class	70	81.4% (72.9–88.6)	0.523 (0.329–0.704)	87.4% (81.2–92.7)	64.9% (50.0–78.0)

Table S15. Full subtype-specific rating distributions.

Rater	Condition	Subtype	Mean (SD); 95% CI	Median [IQR]	≥2
Independent	Real reference	Acinar cell carcinoma	1.40 (1.17); 95% CI 0.70–2.10	1.50 [0.25, 2.00]	5/10 (50.0%)
Independent	Real reference	Microcystic serous cystadenoma	1.40 (0.84); 95% CI 0.90–1.80	2.00 [1.00, 2.00]	6/10 (60.0%)
Independent	Real reference	Solid pseudopapillary neoplasm	1.10 (1.10); 95% CI 0.50–1.70	1.00 [0.00, 2.00]	4/10 (40.0%)
Independent	Real reference	Tubular adenocarcinoma	2.10 (1.10); 95% CI 1.40–2.70	2.50 [1.25, 3.00]	7/10 (70.0%)
Independent	Real reference	Undifferentiated carcinoma	1.70 (1.16); 95% CI 1.00–2.40	2.00 [1.00, 2.75]	6/10 (60.0%)
Independent	Real reference	Undifferentiated carcinoma with osteoclast-like giant cells	1.90 (1.20); 95% CI 1.20–2.60	2.00 [1.25, 3.00]	7/10 (70.0%)
Independent	Real reference	Well-differentiated pancreatic neuroendocrine tumor	1.80 (0.79); 95% CI 1.30–2.20	2.00 [2.00, 2.00]	8/10 (80.0%)
Independent	No truncation	Acinar cell carcinoma	1.50 (1.18); 95% CI 0.80–2.20	1.00 [1.00, 2.75]	4/10 (40.0%)
Independent	No truncation	Microcystic serous cystadenoma	2.00 (0.67); 95% CI 1.60–2.40	2.00 [2.00, 2.00]	8/10 (80.0%)
Independent	No truncation	Solid pseudopapillary neoplasm	1.30 (1.34); 95% CI 0.50–2.10	1.00 [0.00, 2.75]	4/10 (40.0%)
Independent	No truncation	Tubular adenocarcinoma	2.30 (0.48); 95% CI 2.00–2.60	2.00 [2.00, 2.75]	10/10 (100.0%)
Independent	No truncation	Undifferentiated carcinoma	1.20 (1.32); 95% CI 0.40–2.00	1.00 [0.00, 2.00]	5/10 (50.0%)
Independent	No truncation	Undifferentiated carcinoma with osteoclast-like giant cells	0.90 (0.32); 95% CI 0.70–1.00	1.00 [1.00, 1.00]	0/10 (0.0%)
Independent	No truncation	Well-differentiated pancreatic neuroendocrine tumor	1.40 (1.26); 95% CI 0.60–2.10	2.00 [0.00, 2.00]	6/10 (60.0%)
Independent	Per-class	Acinar cell carcinoma	1.90 (0.88); 95% CI 1.40–2.40	2.00 [1.00, 2.75]	6/10 (60.0%)
Independent	Per-class	Microcystic serous cystadenoma	2.90 (0.32); 95% CI 2.70–3.00	3.00 [3.00, 3.00]	10/10 (100.0%)
Independent	Per-class	Solid pseudopapillary neoplasm	2.20 (1.03); 95% CI 1.60–2.80	2.50 [2.00, 3.00]	8/10 (80.0%)

Independent	Per-class	Tubular adenocarcinoma	2.90 (0.32); 95% CI 2.70–3.00	3.00 [3.00, 3.00]	10/10 (100.0%)
Independent	Per-class	Undifferentiated carcinoma	2.40 (0.84); 95% CI 1.90–2.80	3.00 [2.00, 3.00]	8/10 (80.0%)
Independent	Per-class	Undifferentiated carcinoma with osteoclast-like giant cells	1.20 (0.63); 95% CI 1.00–1.60	1.00 [1.00, 1.00]	1/10 (10.0%)
Independent	Per-class	Well-differentiated pancreatic neuroendocrine tumor	2.70 (0.67); 95% CI 2.30–3.00	3.00 [3.00, 3.00]	9/10 (90.0%)
Original	No truncation	Acinar cell carcinoma	1.80 (1.14); 95% CI 1.10–2.40	2.00 [1.25, 2.75]	7/10 (70.0%)
Original	No truncation	Microcystic serous cystadenoma	2.70 (0.48); 95% CI 2.40–3.00	3.00 [2.25, 3.00]	10/10 (100.0%)
Original	No truncation	Solid pseudopapillary neoplasm	1.70 (1.06); 95% CI 1.10–2.30	1.50 [1.00, 2.75]	5/10 (50.0%)
Original	No truncation	Tubular adenocarcinoma	2.90 (0.32); 95% CI 2.70–3.00	3.00 [3.00, 3.00]	10/10 (100.0%)
Original	No truncation	Undifferentiated carcinoma	1.10 (1.10); 95% CI 0.50–1.80	1.00 [0.00, 2.00]	4/10 (40.0%)
Original	No truncation	Undifferentiated carcinoma with osteoclast-like giant cells	0.90 (0.32); 95% CI 0.70–1.00	1.00 [1.00, 1.00]	0/10 (0.0%)
Original	No truncation	Well-differentiated pancreatic neuroendocrine tumor	2.30 (0.67); 95% CI 1.90–2.70	2.00 [2.00, 3.00]	9/10 (90.0%)
Original	Per-class	Acinar cell carcinoma	2.20 (0.42); 95% CI 2.00–2.50	2.00 [2.00, 2.00]	10/10 (100.0%)
Original	Per-class	Microcystic serous cystadenoma	2.90 (0.32); 95% CI 2.70–3.00	3.00 [3.00, 3.00]	10/10 (100.0%)
Original	Per-class	Solid pseudopapillary neoplasm	2.00 (0.47); 95% CI 1.70–2.30	2.00 [2.00, 2.00]	9/10 (90.0%)
Original	Per-class	Tubular adenocarcinoma	3.00 (0.00); 95% CI 3.00–3.00	3.00 [3.00, 3.00]	10/10 (100.0%)
Original	Per-class	Undifferentiated carcinoma	1.30 (0.48); 95% CI 1.00–1.60	1.00 [1.00, 1.75]	3/10 (30.0%)
Original	Per-class	Undifferentiated carcinoma with osteoclast-like giant cells	0.90 (0.32); 95% CI 0.70–1.00	1.00 [1.00, 1.00]	0/10 (0.0%)
Original	Per-class	Well-differentiated pancreatic neuroendocrine tumor	2.50 (0.71); 95% CI 2.10–2.90	3.00 [2.00, 3.00]	9/10 (90.0%)

≥2 indicates that subtype morphology was present.

Section 8. Nearest-Neighbor Copying Screen

Following prior image-copying evaluations that summarize dataset-level behavior using the 95th percentile of each generated image's top-1 training-set similarity [57,58], we performed a nearest-neighbor copying screen. We extracted normalized 512-dimensional CONCH image embeddings [45] for all 70,000 saved synthetic tiles (5000 per subtype and condition) and used exact cosine search to identify the most similar real training tile within the same subtype among all the training tiles. We report the median, interquartile range, 95th percentile (q95), and maximum of the per-image top-1 similarities. Confidence intervals for q95 used 10,000 bootstrap replicates; class-balanced overall intervals were resampled within subtype to preserve equal subtype weights. For the ten highest-similarity pairs per subtype and condition, we additionally evaluated decoded-pixel equality, structural similarity (SSIM), peak signal-to-noise ratio (PSNR), and pixel root mean squared error (RMSE).

The class-balanced q95 was 0.96875 (95% CI, 0.96854–0.96894) for $\psi = 0.5$ and 0.96097 (95% CI, 0.96061–0.96135) for $\psi = 1.0$ (Table S16). The q95 was higher with $\psi = 0.5$ for six subtypes and lower for acinar cell carcinoma, while the single maximum similarity was higher with $\psi = 1.0$ (0.99130 versus 0.98787). Future studies should use a set of real, held-out validation images to benchmark the similarity against the current q95 score to better understand whether the synthetic images are more memorized than unseen real tiles.

Among the 140 reviewed highest-similarity pairs, none was identical in decoded RGB pixels: maximum SSIM was 0.412, median SSIM was 0.075, minimum RMSE was 30.17, and maximum PSNR was 18.54 dB (Figure S10). But SSIM, RMSE and PSNR are strictly spatially aligned metrics; thus, they would fail to flag copied images with slight translations or color shifts.

Table S16. CONCH top-1 training-tile similarity distributions for saved synthetic images.

Condition	Subtype	n	Median	IQR	q95 (95% CI)	Maximum
ψ = 0.5	Overall (class-balanced)	35000	0.94100	0.92077–0.95689	0.96875 (0.96854–0.96894)	0.98787
ψ = 0.5	acinar cell carcinoma	5000	0.92236	0.90540–0.93696	0.95432 (0.95359–0.95505)	0.97554
ψ = 0.5	microcystic serous cystadenoma	5000	0.94432	0.93126–0.95572	0.96750 (0.96680–0.96803)	0.98198
ψ = 0.5	solid pseudopapillary neoplasm	5000	0.95154	0.93555–0.96274	0.97328 (0.97272–0.97385)	0.98787
ψ = 0.5	tubular adenocarcinoma	5000	0.96174	0.95599–0.96622	0.97151 (0.97126–0.97181)	0.98162
ψ = 0.5	undifferentiated carcinoma	5000	0.92418	0.90642–0.93791	0.95322 (0.95240–0.95411)	0.97506
ψ = 0.5	undifferentiated carcinoma with osteoclast-like giant cells	5000	0.93033	0.90977–0.94389	0.95980 (0.95885–0.96051)	0.97829
ψ = 0.5	well-differentiated pancreatic neuroendocrine tumor	5000	0.94588	0.92793–0.95814	0.96936 (0.96886–0.96997)	0.98356
ψ = 1.0	Overall (class-balanced)	35000	0.91483	0.88100–0.93912	0.96097 (0.96061–0.96135)	0.99130
ψ = 1.0	acinar cell carcinoma	5000	0.89855	0.86157–0.92758	0.95681 (0.95542–0.95796)	0.97913
ψ = 1.0	microcystic serous cystadenoma	5000	0.92200	0.89357–0.94296	0.96259 (0.96167–0.96339)	0.98174
ψ = 1.0	solid pseudopapillary neoplasm	5000	0.91941	0.88611–0.94383	0.96757 (0.96639–0.96814)	0.99130
ψ = 1.0	tubular adenocarcinoma	5000	0.93861	0.92020–0.95162	0.96365 (0.96307–0.96423)	0.97667
ψ = 1.0	undifferentiated carcinoma	5000	0.90048	0.86841–0.92392	0.94730 (0.94609–0.94850)	0.97254
ψ = 1.0	undifferentiated carcinoma with osteoclast-like giant cells	5000	0.89214	0.85423–0.92187	0.94992 (0.94865–0.95125)	0.97913
ψ = 1.0	well-differentiated pancreatic neuroendocrine tumor	5000	0.92054	0.89309–0.94221	0.96252 (0.96153–0.96328)	0.98005

q95 confidence intervals are percentile 95% bootstrap intervals from 10,000 replicates; overall intervals preserve equal subtype weights.

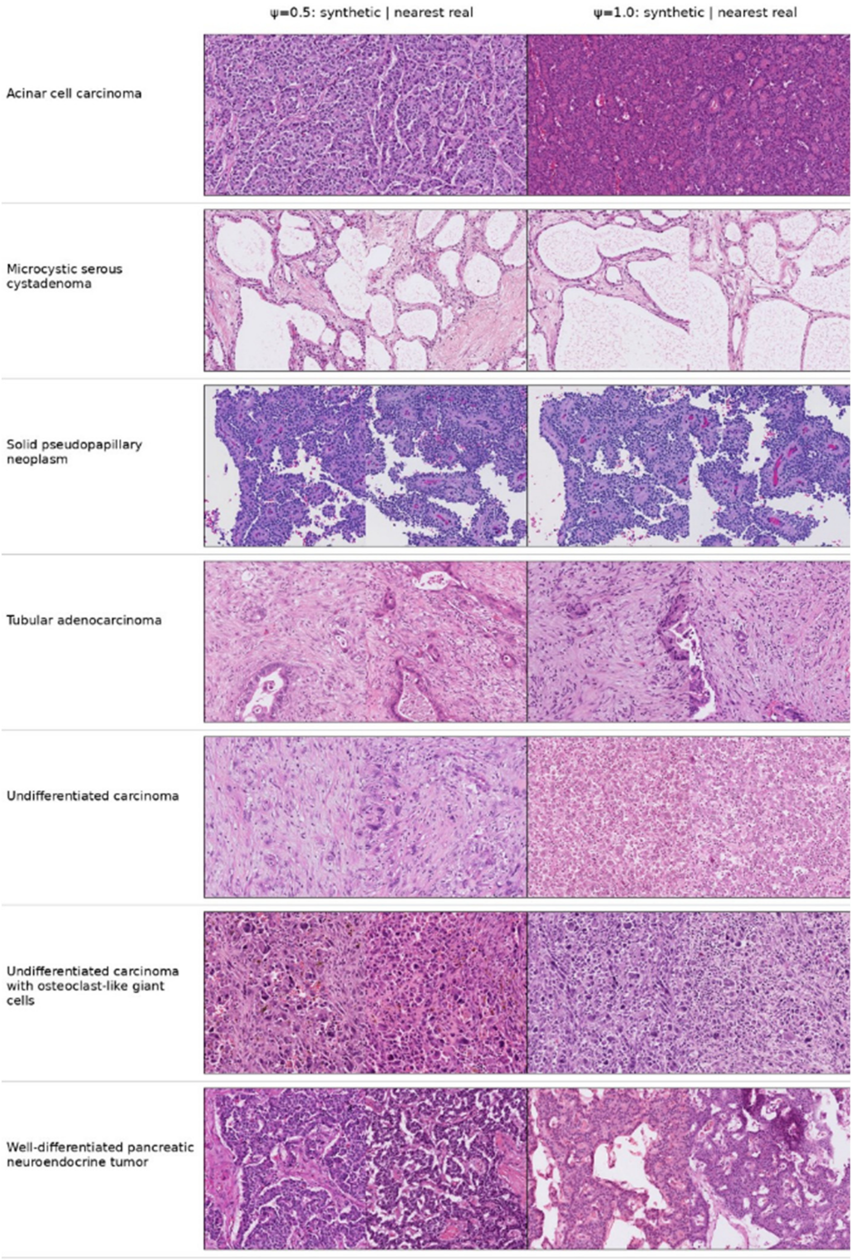

Figure S10. Highest-CONCH-similarity synthetic/training pair for each subtype and truncation condition. Within each condition block, the synthetic image is shown on the left and its nearest real training tile on the right. None of the displayed pairs was identical in decoded RGB pixels.

Section 9. Independent-Cohort Evaluation of Real-Only and Synthetic-Augmented Subtype Classifiers

To assess whether synthetic images improved downstream tumor-subtype classification, we trained two seven-class ResNet50 classifiers: one using real tiles only and one using real tiles augmented with per-class-truncated StyleGAN2 images. Both models were initialized from the same ImageNet-pretrained checkpoint and received each complete 1024 × 1024 tile resized to 512 × 512 pixels. The internal development cohort was divided by case into 394 training and 98 validation cases: all tiles from a case remained within one partition. Real training samples were drawn by selecting the subtype uniformly, then a case within that subtype uniformly, and finally a tile within that case uniformly. This prevented cases with many tiles from dominating training.

For the augmented model, we used 35,000 synthetic images generated with per-class truncation ($\psi = 0.5$; 5000 images per subtype). Synthetic samples were drawn uniformly by subtype. At each optimizer update, the augmented objective was 0.75 x real cross-entropy + 0.25 x synthetic cross-entropy, corresponding to a 3:1 real:synthetic loss

contribution. Both arms used the same case split, initialization seed, batch size (32), AdamW optimizer (learning rate = 10^{-4} and weight decay = 10^{-4}), cosine learning-rate schedule, mixed-precision training, early stopping, and a maximum of eight epochs. Each arm independently selected its checkpoint using case-aggregated balanced accuracy on the internal validation cases, with patience of two epochs. Internal validation was used only for checkpoint selection and was not interpreted as independent evidence of augmentation benefit because the image generator had been trained using the complete internal cohort.

The trained models were evaluated once on an independent test cohort from Johns Hopkins Hospital comprising 205 tiles from 40 patients, with one slide per patient. These patients were independent of the training cohort, and the tiles were acquired at the same 0.5 mpp as the training data. The cohort included all seven modeled subtypes. Because the development and test cohorts originated from the same hospital, this experiment assessed generalization to unseen patients but did not constitute external institutional validation.

We report case-level F1 and one-vs-rest area under the receiver operating characteristic curve (AUROC) for each subtype. Across subtypes, macro-F1 increased from 0.236 (95% CI 0.131–0.333) for the real-only model to 0.445 (0.350–0.530) for the synthetic-augmented model. Macro-AUROC increased from 0.758 (0.677–0.838) to 0.829 (0.769–0.882), with a paired difference of 0.071 (-0.014 to 0.153). The largest concordant improvement in F1 and AUROC was observed for well-differentiated pancreatic neuroendocrine tumor. Undifferentiated carcinoma also improved on both metrics. Effects were not uniform across subtypes: for example, solid pseudopapillary neoplasm showed higher AUROC but lower F1 after augmentation, indicating improved one-vs-rest ranking without improved final multiclass assignment. Acinar cell carcinoma had F1 of zero under both models despite AUROC above 0.80, likewise demonstrating that ranking performance did not translate into correct classification.

These findings provide independent same-institution evidence that the evaluated synthetic-augmentation regimen can improve overall subtype classification, but they do not establish a benefit for every subtype. In addition, the test cohort was also small and unevenly distributed: microcystic serous cystadenoma was represented by one case.

Table S17. Case-level F1 and one-vs-rest AUROC for real-only and synthetic-augmented classifiers on the independent same-institution test cohort. .

Subtype	n	Real only		Real + synthetic	
		F1	AUROC	F1	AUROC
Acinar cell carcinoma	5	0.00 (0.00, 0.00)	0.82 (0.67, 0.94)	0.00 (0.00, 0.00)	0.86 (0.69, 0.99)
Microcystic serous cystadenoma	1	0.00 (0.00, 0.00)	1.00 (1.00, 1.00)	1.00 (1.00, 1.00)	1.00 (1.00, 1.00)
Solid pseudopapillary neoplasm	6	0.44 (0.00, 0.80)	0.63 (0.39, 0.86)	0.29 (0.00, 0.67)	0.95 (0.88, 1.00)
Tubular adenocarcinoma	5	0.50 (0.27, 0.71)	0.89 (0.77, 0.97)	0.60 (0.22, 0.91)	0.78 (0.51, 1.00)
Undifferentiated carcinoma	7	0.32 (0.10, 0.56)	0.62 (0.39, 0.84)	0.48 (0.41, 0.56)	0.84 (0.71, 0.94)
Undifferentiated carcinoma with osteoclast-like giant cells	3	0.15 (0.00, 0.40)	0.63 (0.35, 0.87)	0.00 (0.00, 0.00)	0.45 (0.33, 0.57)
Well-differentiated pancreatic neuroendocrine tumor	13	0.24 (0.00, 0.50)	0.72 (0.50, 0.90)	0.75 (0.53, 0.92)	0.92 (0.83, 0.98)

Values are estimates with percentile 95% confidence intervals from 10,000 paired, subtype-stratified case-bootstrap replicates. The augmented model used per-class-truncated synthetic images (ψ = 0.5) with a 3:1 real:synthetic loss contribution. n is the number of independent test cases with the indicated subtype.
